# Coordinated activation of distinct Ca^2+^ sources and metabotropic glutamate receptors encodes Hebbian synaptic plasticity

**DOI:** 10.1038/ncomms10289

**Published:** 2016-01-13

**Authors:** Cezar M. Tigaret, Valeria Olivo, Josef H.L.P. Sadowski, Michael C. Ashby, Jack R. Mellor

**Affiliations:** 1 Centre for Synaptic Plasticity, School of Physiology, Pharmacology and Neuroscience, University of Bristol, University Walk, Bristol BS8 1TD, UK

## Abstract

At glutamatergic synapses, induction of associative synaptic plasticity requires time-correlated presynaptic and postsynaptic spikes to activate postsynaptic NMDA receptors (NMDARs). The magnitudes of the ensuing Ca^2+^ transients within dendritic spines are thought to determine the amplitude and direction of synaptic change. In contrast, we show that at mature hippocampal Schaffer collateral synapses the magnitudes of Ca^2+^ transients during plasticity induction do not match this rule. Indeed, LTP induced by time-correlated pre- and postsynaptic spikes instead requires the sequential activation of NMDARs followed by voltage-sensitive Ca^2+^ channels within dendritic spines. Furthermore, LTP requires inhibition of SK channels by mGluR1, which removes a negative feedback loop that constitutively regulates NMDARs. Therefore, rather than being controlled simply by the magnitude of the postsynaptic calcium rise, LTP induction requires the coordinated activation of distinct sources of Ca^2+^ and mGluR1-dependent facilitation of NMDAR function.

## Abstract (web summary)

During STDP, the magnitude of postsynaptic Ca^2+^ transients is hypothesized to determine the strength of synaptic plasticity. Here, the authors find that STDP in mature hippocampal synapses does not obey this rule but instead relies on the coordinated activation of NMDARs and VGCCs and their regulation by mGluRs and SK channels.

## Graphical abstract

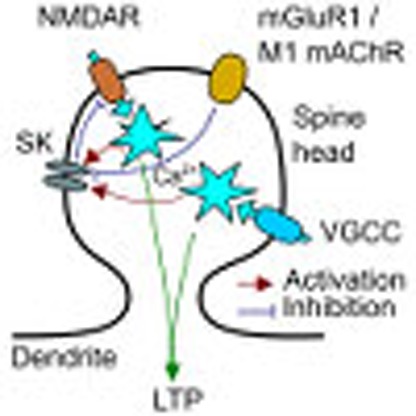

Hebbian synaptic plasticity at glutamatergic synapses mediated by NMDA receptors (NMDARs) is the principal mechanism underlying associative learning. The classical exposition of Hebbian plasticity is spike timing-dependent plasticity (STDP) where temporally correlated pre- and postsynaptic activity induces bidirectional NMDAR- and Ca^2+^-dependent changes in synaptic strength^1^. Canonical STDP occurs when a presynaptic spike is paired with a postsynaptic action potential and has two defining characteristics: (1) the magnitude of synaptic plasticity is inversely related to the millisecond delay between the pre- and the postsynaptic spikes and (2) the direction of plasticity is determined by the temporal order of the spikes, with pre-before-post leading to long-term potentiation (LTP) and post-before-pre leading to long-term depression (LTD)^2^,^3^,^4^.

The Ca^2+^ hypothesis for synaptic plasticity as applied to STDP states that the activation of synaptic NMDARs and the ensuing excitatory postsynaptic Ca^2+^ transients (EPSCaTs) within dendritic spines provide a critical trigger for the induction of plasticity, with large EPSCaTs leading to LTP whereas moderate signals induce LTD (Fig. 3a)^5^,^6^,^7^. In this framework the postsynaptic spike provides the necessary depolarization for removal of NMDAR block by Mg^2+^ within a narrow time window relative to synaptic release of glutamate^8^,^9^ (but see ref. 10).

Evidence in support of the Ca^2+^ hypothesis comes from three main sources. First, synaptic plasticity is voltage dependent; pairing of synaptic stimulation with moderate depolarization induces LTD whereas pairing with strong depolarization induces LTP^11^,^12^. Second, titration of EPSCaTs with increasing concentrations of postsynaptic Ca^2+^ chelators shapes the induction of plasticity according to Ca^2+^ hypothesis predictions^12^,^13^,^14^. Third, the magnitude of somatic/dendritic postsynaptic Ca^2+^ transients correlates with the direction and amplitude of synaptic plasticity^15^,^16^,^17^. However, this correlation may not apply to EPSCaTs within dendritic spines. Most dendritic spines behave as semi-independent biochemical compartments endowed with intrinsic mechanisms for controlling the local concentration of free Ca^2+^ (refs 18, 19, 20). The electrical compartmentalization provided by the spine neck^21^,^22^ can further shape the local spine Ca^2+^ signals triggered by synaptic activation^23^,^24^. Since it is proposed that high Ca^2+^ buffering capacity within spines localizes Ca^2+^ signals to nanodomains surrounding Ca^2+^ permeable channels^25^,^26^,^27^, Ca^2+^ from distinct spatial locations may activate specific molecular events to express LTP or LTD. Indeed, the correlation between EPSCaT magnitude and synaptic plasticity breaks down when applied to STDP^28^ presenting a potential flaw in the Ca^2+^ hypothesis. In this instance the breakdown in correlation may be due to NMDAR-independent STD–LTD at immature synapses^28^,^29^,^30^,^31^ but this still leaves open the mechanisms governing STDP at mature synapses.

We therefore sought to test the Ca^2+^ hypothesis for synaptic plasticity by correlating the magnitude of EPSCaT during STDP induction with the magnitude and direction of synaptic plasticity at mature Schaffer collateral synapses in the hippocampus. We find that EPSCaT magnitude alone cannot accurately predict the induction of synaptic plasticity and instead STDP requires precisely timed Ca^2+^ transients mediated by NMDARs and voltage-sensitive Ca^2+^ channels (VSCCs). In addition, STDP is facilitated by activation of mGlu1 receptors that inhibit SK channels that otherwise restrict NMDAR opening. Collectively, our data suggest that the requirement for postsynaptic spikes in the induction of plasticity at mature CA3-CA1 synapses is explained by the coordinated activation of distinct spine Ca^2+^ sources and of mGluR1.

## Results

### STDP at mature hippocampal synapses

**Figure 1:**
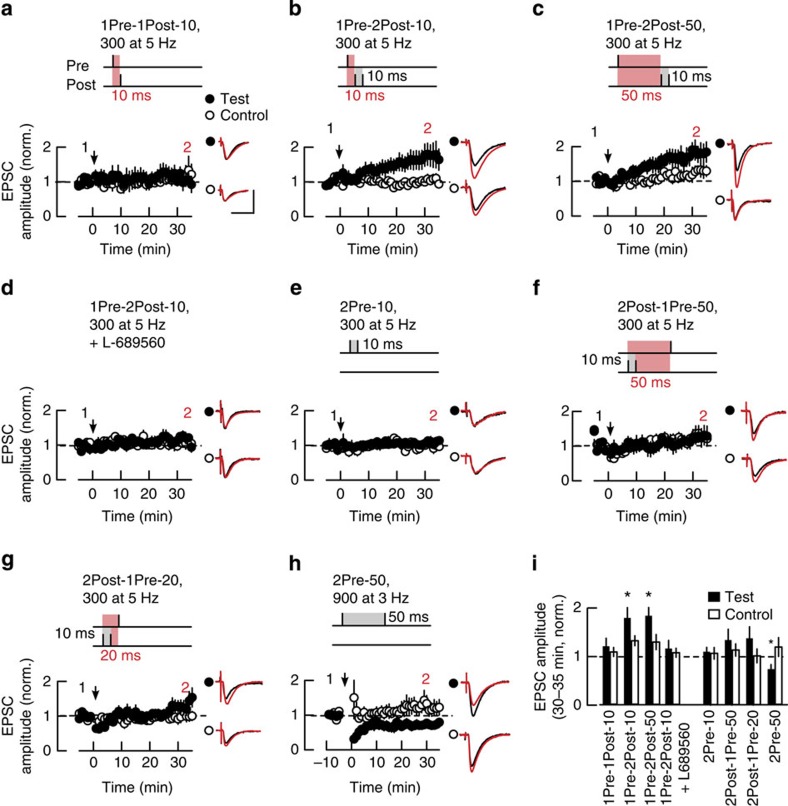
Electrophysiological rules for induction of pathway-specific LTP at mature Schaffer collateral–CA1 pyramidal neuron synapses. (**a**–**h**) LTP induction by theta frequency trains requires causal association of synaptic input with postsynaptic activity bursts. (**a**) Single presynaptic EPSPs paired with single bAPs at 10 ms interval fails to induce plasticity (1Pre-1Post-10; *n*=6). (**b**,**c**) Single EPSPs paired with two bAPs at 10 ms interval (1Pre-2Post-10, **b**; *n*=8) or at 50 ms interval (1Pre-2Post-50, **c**; *n*=7) induce test pathway-specific LTP. (**d**) NMDAR antagonist L-689560 (5 μM, *n*=5) blocks induction of LTP by a train of 1Pre-2Post-10 stimulations. (**e**) Presynaptic paired pulses at 10 ms interval do not induce plasticity (2Pre-10; *n*=8). (**f**,**g**). Single EPSPs preceded by two bAPs at 50 ms (2Post-1Pre-50, **f**; *n*=6) or 20 ms interval (2Post-1Pre-20, **g**; *n*=10) do not induce plasticity. (**h**) Paired EPSPs at 50 ms interval (3 Hz, 5 min) induce test pathway-specific LTD (2Pre-50; *n*=9). Plots in **a**–**h** show the time course of the EPSC amplitude in test and control pathways normalized to the 5 min average before plasticity induction protocol was applied to the test pathway (arrows). Top schemes depict the stimulation protocol used in each panel. Insets: average EPSC waveforms before (1, black) and 30–35 min after plasticity induction (2, red) recorded in test and control pathways. Scale bars, 50 pA and 50 ms. (**i**) Summary of changes in normalized EPSC amplitude at 30–35 min after the induction protocols in **a**–**h**. *P<0.05, Wilcoxon rank-sum test.

To test the Ca^2+^ hypothesis for STDP we sought to determine whether EPSCaT amplitude predicts the magnitude and direction of synaptic plasticity in response to STDP induction protocols. We first characterized the plasticity induction rules at adult Schaffer collateral-CA1 synapses by defining a range of stimulation protocols pairing subthreshold presynaptic stimuli (excitatory postsynaptic potentials (EPSPs)) with postsynaptic back-propagating action potentials (bAPs) and elicited as a causal (EPSP-bAP) or anti-causal (bAP-EPSP) pairing (referred to as Pre–Post or Post–Pre pairings, respectively) over a range of time intervals (Fig. 1a–c,f,g,i and Methods section). The stimulations were applied to the test pathway as a single theta frequency train (300 simulations at 5 Hz, for 1 min)^32^. Causal pairing of single EPSPs with single bAPs at 10 ms interval (1Pre-1Post-10) failed to induce plasticity (Fig. 1a,i). However, robust pathway-specific LTP was induced when two bAPs were elicited 10–50 ms after the onset of the EPSP (Fig. 1b,c,i). This confirmed results showing developmental changes in the activity requirements for synaptic plasticity at hippocampal Schaffer collateral-CA1 synapses where induction of LTP at mature synapses requires pairing presynaptic activity with a burst of postsynaptic action potentials^32^,^33^. In common with other STDP studies^3^,^32^,^33^,^34^ LTP was expressed gradually over a period of 20–30 min reflecting the absence of an initial post-tetanic potentiation, which is more characteristic of induction protocols that use high-frequency presynaptic stimulation (Supplementary Fig. 1).

LTP induced by the 1Pre-2Post-10 stimulation protocol was blocked by the NMDAR Gly-site antagonist L-689560 (5 μM) confirming that this form of synaptic plasticity is NMDAR dependent (Fig. 1d,i). In addition, consistent with the requirement of postsynaptic spikes for induction of LTP, presynaptic activity alone delivered as a train of paired subthreshold EPSPs at 10 ms inter-spike interval (ISI) did not induce plasticity (Fig. 1e,i).

The canonical STDP paradigm posits that the temporal order of pre- and postsynaptic spikes determines the direction of plasticity^2^,^3^. We tested this hypothesis and found that anti-causal association of one EPSP with two bAPs failed to induce plasticity irrespective of the time interval (Fig. 1f,g,i). Since none of the induction protocols tested so far generated any LTD, we asked whether synaptic depression could be induced in our preparations. Indeed, a train of 900 presynaptic pulse pairs at 50 ms ISI delivered at 3 Hz for 5 min^34^ induced a robust LTD (Fig. 1h,i). This suggests that the cellular mechanisms for synaptic depression are functional at these synapses, although not engaged by the bAP-EPSP spike timing protocols tested.

Our results show a requirement for minimal postsynaptic activity bursts to induce Hebbian LTP at adult Schaffer collateral-CA1 synapses, in agreement with previous reports^32^,^33^,^35^. Furthermore, our data show that anti-causal spike pairing does not induce LTD and that instead prolonged low-frequency stimulation is required.

### Comparison of spine EPSCaTs with STDP

**Figure 2:**
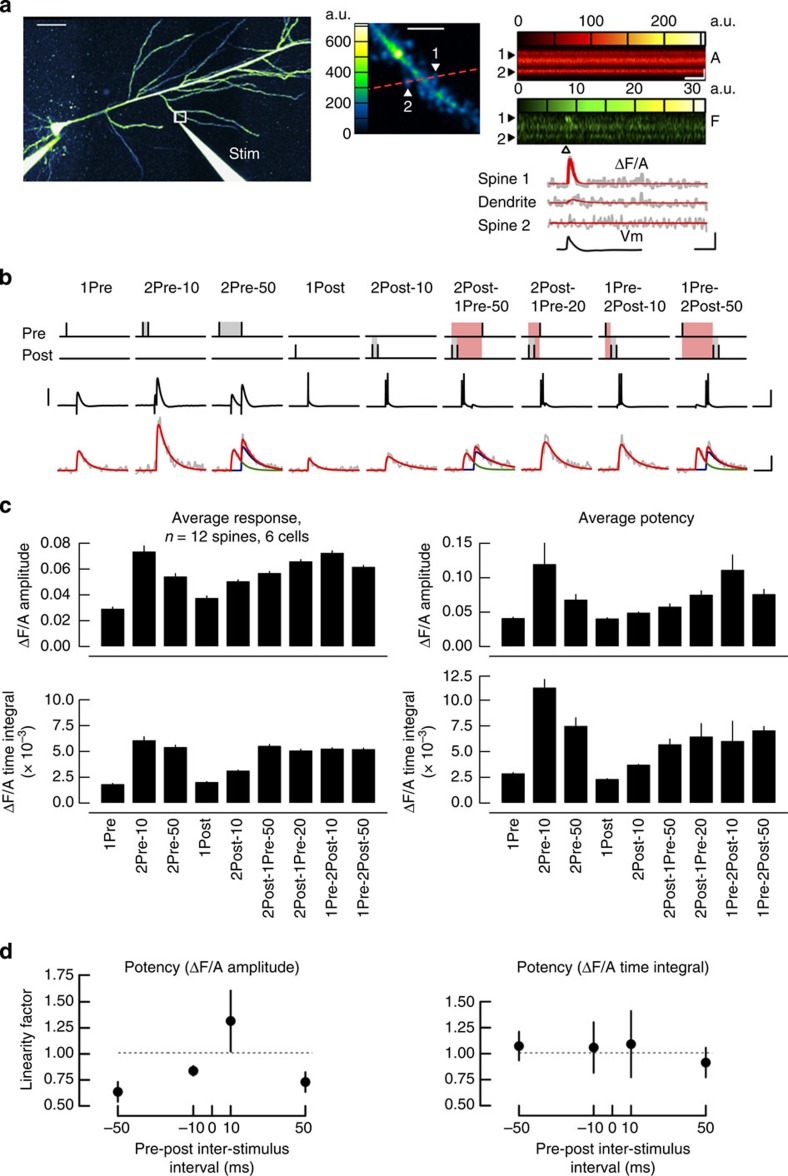
Magnitude of spine EPSCaTs elicited by pre- and postsynaptic spike pairing. (**a**) Two-photon line-scan imaging of spine EPSCaTs. Left: pseudo-colour overview of a CA1 pyramidal cell patch-loaded with Fluo-5F (F) and Alexa Fluor 594 (A) visualized in the Alexa channel. Stim: Alexa-filled extracellular patch electrode in stratum radiatum, near an oblique apical dendrite. White square marks the region scaled up in right top. Scale bar, 50 μm. Right top: two spines (1, 2; filled arrowheads) and their parent dendrite were imaged in line-scan mode (red dashed line shows the scan direction). Scale bar, 5 μm. Right middle: dual-channel (A and F) visualization of a line-scan series (time on the abscissa), through spines 1 and 2; a single EPSP was elicited (arrowhead) via the Stim electrode. Right bottom: the corresponding EPSCaT traces (ΔF/A, grey and double exponential fit, red) for spines 1 and 2 and parent dendrite, and the somatic membrane potential (Vm) recorded during the stimulus. Scale bars, horizontal: 0.1 s; vertical: 2 μm and 0.05 ΔF/A. a.u., arbitrary units. (**b**) Somatic membrane potentials (middle) and EPSCaT waveforms (bottom, grey) evoked by the stimulations depicted at top (see also Fig. 1). Traces are averages of 7–9 trials. 2Pre-50, 2Post-1Pre-50 and 1Pre-2Post-50 EPSCaTs were fitted with two double exponential curves (blue and green), which were then summated (red). Scale bars, horizontal: 0.1 s; vertical: 50 mV (10 mV for 1Pre, 2Pre-10 and 2Pre-50) and 0.05 ΔF/A for EPSCaTs. (**c**) Summary of EPSCaT amplitudes and time integrals for the stimulations in **b**. Left: average values across all trials (including failures). Right: average potencies (see Methods section). (**d**) Linearity factors for the summation of EPSCaT amplitude and time integral evoked by pre–post paired stimulations, versus time interval between the onset of EPSP and the first bAP (red in the schematics of panel **b**). Data calculated from the potencies in panel **c** (right) and Supplementary Fig. 4b.

A direct test of the Ca^2+^ hypothesis for STDP is to correlate the magnitude of the Ca^2+^ transients (EPSCaTs) elicited by paired stimulations in individual dendritic spines with the change in synaptic efficacy. To determine this correlation we performed dual-channel two-photon imaging of Ca^2+^ transients in individual spines located on oblique branches of apical dendrites, where the majority of the excitatory Schaffer collateral input arises. Synapses on spines located 120–280 μm from the soma (Supplementary Fig. 2) were stimulated locally via an extracellular electrode and bAPs were evoked by current injections via the somatic patch electrode (Fig. 2a and Methods section). Our aim was to compare the EPSCaTs evoked at single spines by the paired stimulations tested in the LTP experiments. We performed EPSCaT imaging separately from the LTP experiments to avoid potential LTP washout during dye loading of the cells in whole-cell mode and employed the same physiological conditions. We interleaved different stimulation protocols at a low frequency (0.05–0.1 Hz) at the same synapse to directly compare responses while minimizing photodamage. To test the validity of comparing the low-frequency stimulation with the 5-Hz stimulation used in LTP experiments we also imaged spine Ca^2+^ during the 60 s 5 Hz stimulus trains. EPSCaTs did not summate and attenuated rapidly, with the largest EPSCaTs occurring within the first second of the 5 Hz stimulation (Supplementary Fig. 3a–c). This attenuation was likely due to a steady-state EPSP attenuation (Supplementary Fig. 3d) combined with activity-dependent depression of action potential back-propagation and voltage-gated calcium channels^36^,^37^,^38^,^39^. Thus EPSCaTs evoked by individual stimuli at low frequency accurately represent the spine Ca^2+^ signals elicited during the effective time window of the LTP induction protocol. To further reduce the amount of light excitation we separated the stimulation protocols into two groups and tested them in separate experiments (Fig. 2 and Supplementary Fig. 4a,b). The analysis aggregates data from both sets of experiments where there was overlap.

Single subthreshold EPSPs (1Pre) evoked EPSCaTs with average ΔF/A amplitude and time integral, respectively, of 0.029±0.004 and (3.8±0.7) × 10^−3^, rising to 0.080±0.008 (*P*<0.001) and (14.2±2.2) × 10^−3^ (*P*<0.001, both compared with 1Pre, one-way analysis of variance (ANOVA) with *post hoc* Tukey HSD test) when triggered by a pair of subthreshold EPSPs at 10 ms interval (2Pre-10). Normalizing these values to the EPSCaTs evoked by 1Pre for each spine revealed that the Ca^2+^ transients elicited by 2Pre-10 summated supra-linearly (ΔF/A amplitude and time integral, respectively, of 3.5±0.43 and (7.8±1.9) × 10^−3^, both *P*<0.001, Wilcoxon rank-sum test). Single bAPs (1Post) elicited Ca^2+^ transients with average ΔF/A amplitude of 0.026±0.004, and time integral of (2.9±0.4) × 10^−3^ that summated to 0.042±0.004 and (4.9±0.67) × 10^−3^, respectively, when elicited with two bAPs at 10 ms ISI (2Post).

EPSCaTs evoked by paired pre- and postsynaptic stimulations were either smaller or not significantly different from those evoked by 2Pre-10 (Fig. 2b,c and Supplementary Fig. 4a,b). In particular, the LTP-inducing 1Pre-2Post-10 and 1Pre-2Post-50 stimulations or the canonical STDP simulation 1Pre-1Post-10 evoked smaller EPSCaTs compared with 2Pre-10 (ΔF/A amplitude and time integral, respectively, for 1Pre-2Post-10: 0.066±0.006, *P*=0.35, and (9.7±1.5) × 10^−3^, *P*<0.05; 1Pre-2Post-50: 0.047±0.019, *P*<0.05, and (4.7±2) × 10^−3^, *P*<0.001; 1Pre-1Post-10: 0.052±0.006, *P*<0.01, and (8.4±1.4) × 10^−3^ , *P*<0.05; one-way ANOVA with *post hoc* Dunnett test).

Presynaptic stimuli elicited EPSCaTs stochastically with an apparent success rate for 1Pre of 0.68±0.05 rising to 0.93±0.02 for 2Pre-10, and to 0.88±0.04 for 2Pre-50 stimulations, reflecting failures in neurotransmitter release^40^,^41^. We also observed failures in spine Ca^2+^ transients evoked by 1Post and 2Post (success rates 0.74±0.05 and 0.86±0.03, respectively), consistent with previous observations^42^. Pairing pre- and postsynaptic stimuli raised the apparent success rates to 0.98±0.01 (2Post-1Pre-50), 0.98±0.01 (2Post-1Pre-20), 0.87±0.04 (1Pre-1Post-10), 0.93±0.03 (1Pre-2Post-10) and 0.92±0.04 (1Pre-2Post-50). To avoid the potential bias in efficacy measurement introduced by different success rates when averaging across multiple trials, we compared the potencies of EPSCaTs evoked by the various stimulations. For single stimuli and paired stimulations with 50 ms ISI the EPSCaT potencies were determined directly by averaging successful trials, whereas for paired stimulations with 10 ms ISI they were inferred as described in Methods section (Fig. 2c and Supplementary Fig. 4b). The potencies of Ca^2+^ transients elicited by paired pre- and postsynaptic stimulations were smaller compared with the 2Pre-10 EPSCaTs. The ΔF/A amplitude and time integral potencies were, respectively, for 2Pre-10: 0.10±0.01 and (18.7±2.6) × 10^−3^; 1Pre-2Post-10: 0.068±0.01, *P*=0.1, and (13.4±2) × 10^−3^, *P*<0.05; 1Pre-2Post-50: 0.064±0.008, *P*<0.05, and (5.8±0.8) × 10^−3^, *P*<0.001; 1Pre-1Post-10: 0.074±0.01, *P*<0.05, and (10±1.6) × 10^−3^, *P*<0.001; all *P*-values compared 2Pre-10 potencies using one-way ANOVA and *post hoc* Dunnett test). The observation that LTP-inducing stimulations evoke smaller EPSCaTs relative to 2Pre-10 was consistent across the entire range of spines and was not correlated with the magnitude of EPSCaTs evoked by a single presynaptic stimulation (Supplementary Fig. 5). In addition, EPSCaTs evoked by the LTD-inducing stimulation 2Pre-50 had ΔF/A amplitude 0.054±0.008 and time integral (5.4±0.7) × 10^−3^. These analyses reveal the relative potency of EPSCaTs elicited by the different stimulation protocols and confirm that pairs of subthreshold EPSPs that do not induce plasticity (2Pre-10) produce the largest EPSCaTs.

The Ca^2+^ transients induced by pairing pre- and postsynaptic spikes have been shown in some cortical and hippocampal preparations *in vitro* to reflect a supra-linear summation of the Ca^2+^ signals evoked by the individual spikes delivered separately^43^,^44^,^45^, but see ref. 46. Indeed, the potency of EPSCaTs triggered by 1Pre-2Post pairings summated supra-linearly at 10 ms ISI and became sub-linear for anti-causal pairings or longer causal intervals (Fig. 2d). The reported variability in supra-linearity could be due to a number of factors including the age of animals or resting membrane potential of recorded cells.

**Figure 3:**
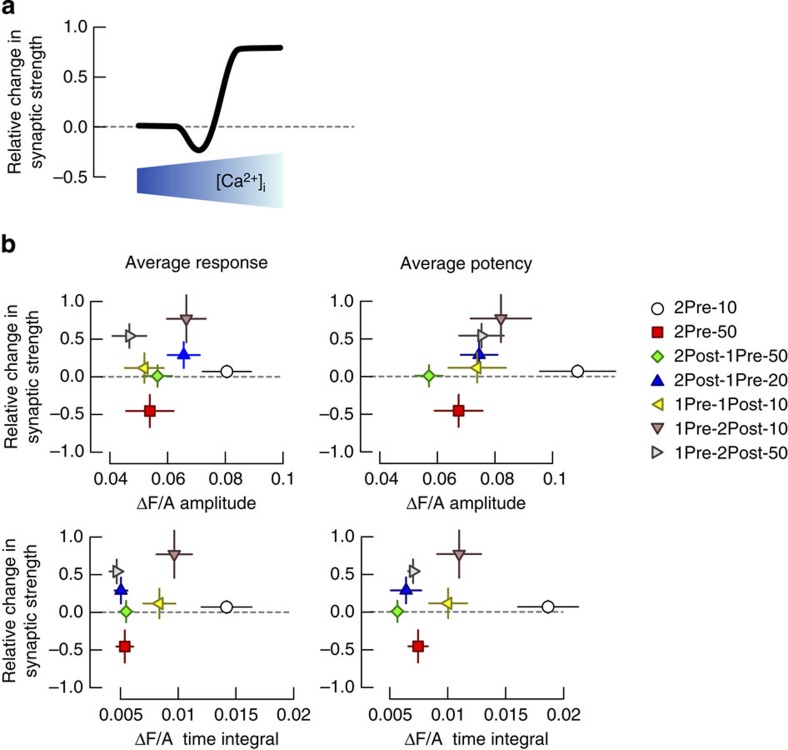
The electrophysiological rules for induction of plasticity do not match the size of spine EPSCaTs triggered by paired pre- and postsynaptic spikes. (**a**) Hypothesized relationship between the relative change in synaptic strength and free Ca^2+^ concentration during a stimulation-evoked Ca^2+^ transient in the spine. (**b**) Relationship between the relative change in synaptic strength for data in Fig. 1 and amplitude (top) or time integral (bottom) for the average EPSCaT response (left) or potency (right). The EPSCaT data are summaries of experiments in Fig. 2 and Supplementary Fig. 4.

To summarize the relationship between EPSCaTs and plasticity induction by the various stimulation protocols, we compared the EPSCaT amplitude and time integral values to the change in synaptic strength (Methods section). Since the EPSCaT magnitude was recorded separately (and in separate spines) from the plasticity, data were category-binned according to the paired stimulation. As shown in Fig. 3, the relationship bore no immediate comparison to the predictions made by the Ca^2+^ hypothesis for STDP (Fig. 3a). Furthermore there was no linear correlation between the change in synaptic strength and the average EPSCaT magnitude (Pearson correlation coefficient and 95% CI, respectively, for amplitude: 0.08, −0.17 to 0.78, *P*=0.85 and time integral: 0.1, −0.7 to 0.79, *P*=0.82). Similarly, the change in synaptic strength did not correlate with the EPSCaT potency (amplitude: 0.21, −0.63 to 0.83, *P*=0.63; time integral: 0.05, −0.72 to 0.77, *P*=0.91). These results indicate that the magnitude of EPSCaTs evoked by paired pre- and postsynaptic stimulations is not sufficient to explain the plasticity induction rules at mature Schaffer collateral-CA1 synapses.

The critical events required for LTP induction are postsynaptic action potentials occurring within close temporal proximity after presynaptic release of glutamate^2^,^3^. Since we show this requirement is not dependent on the total amount of Ca^2+^ influx into dendritic spines (Fig. 3) we next tested the hypothesis that Ca^2+^ influx through VSCCs activated by bAPs immediately after Ca^2+^ influx through NMDARs is required for LTP.

### Role of VSCCs in STDP

Back-propagated action potentials activate VSCCs at the dendritic spine, such that the bAP-evoked spine Ca^2+^ transients arise mainly from the activation of the Ca_V_3 (T-type), Ca_V_2.3 (R-type) and Ca_V_1.2/1.3 (L-type) VSCCs^47^. VSCCs contribute to LTP induced by high-frequency burst firing of pre- and postsynaptic neurones^48^,^49^ but are not required for STD–LTP in hippocampal cultures or cortical synapses^3^,^28^. We hypothesized that the activation of these VSCC types by bAPs provides a critical signal for LTP induction in the causal pre–post-pairing stimulation.

**Figure 4:**
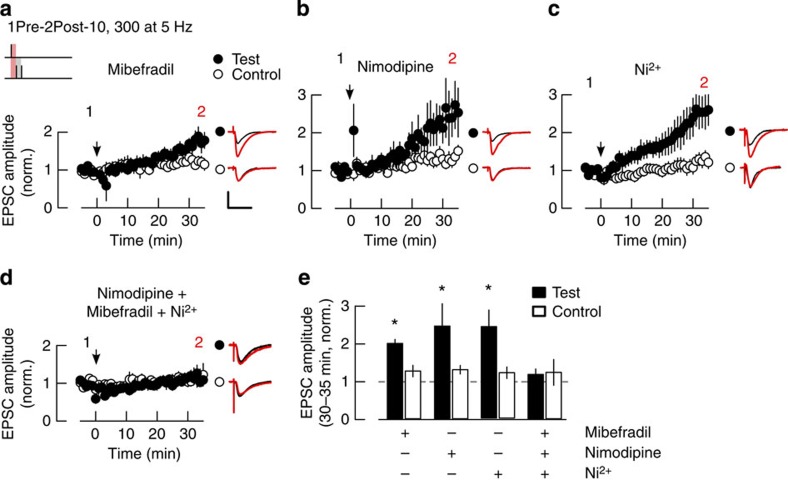
Induction of LTP by paired pre- and postsynaptic spikes requires the activation of NMDARs and VSCCs. (**a**–**c**) LTP induction occurs under the selective block of either T-type (Mibefradil, 5 μM, *n*=7), L-type (Nimodipine 20 μM, *n*=6), or R-type (NiCl_2_ 50 μM, *n*=5) VSCCs. (**d**) The combined block of L-, T- and R-type VSCCs inhibits induction of LTP (drug concentrations as in **a**–**c**, *n*=8). Drugs were bath-applied throughout the experiment. Panels depict the time course of the EPSC amplitude for test and control pathways, normalized to 5 min average before plasticity induction (arrows). Insets show 5 min average EPSC waveforms before (1) and 30–35 min after plasticity induction (2) in test and control pathways. Scale bars: 50 pA and 50 ms. (**e**) Summary of changes in normalized EPSC amplitude 30–35 min after the induction, for the treatments in **a**–**d**. *P<0.05, Wilcoxon rank-sum test.

To determine the contribution of these VSCC types to LTP induction by a train of 1Pre-2Post-10 stimulations we used the T-type-preferring blocker Mibefradil (5 μM), the L-type-selective antagonist Nimodipine (20 μM), and the R-type preferring blocker NiCl_2_ (50 μM)^50^. The block of individual VSCC types did not affect LTP induction (Fig. 4a–c,e) and the magnitudes of LTP were not significantly different from that of the control experiment without drugs. However, collective block of all three VSCC types with a cocktail of antagonists did block LTP (Fig. 4d,e). These results indicate that activation of all three VSCC types contribute to the induction of LTP by 1Pre-2Post-10 stimulation. The fact that individual selective antagonists did not inhibit LTP suggests a compensatory effect by the unblocked VSCC types.

**Figure 5:**
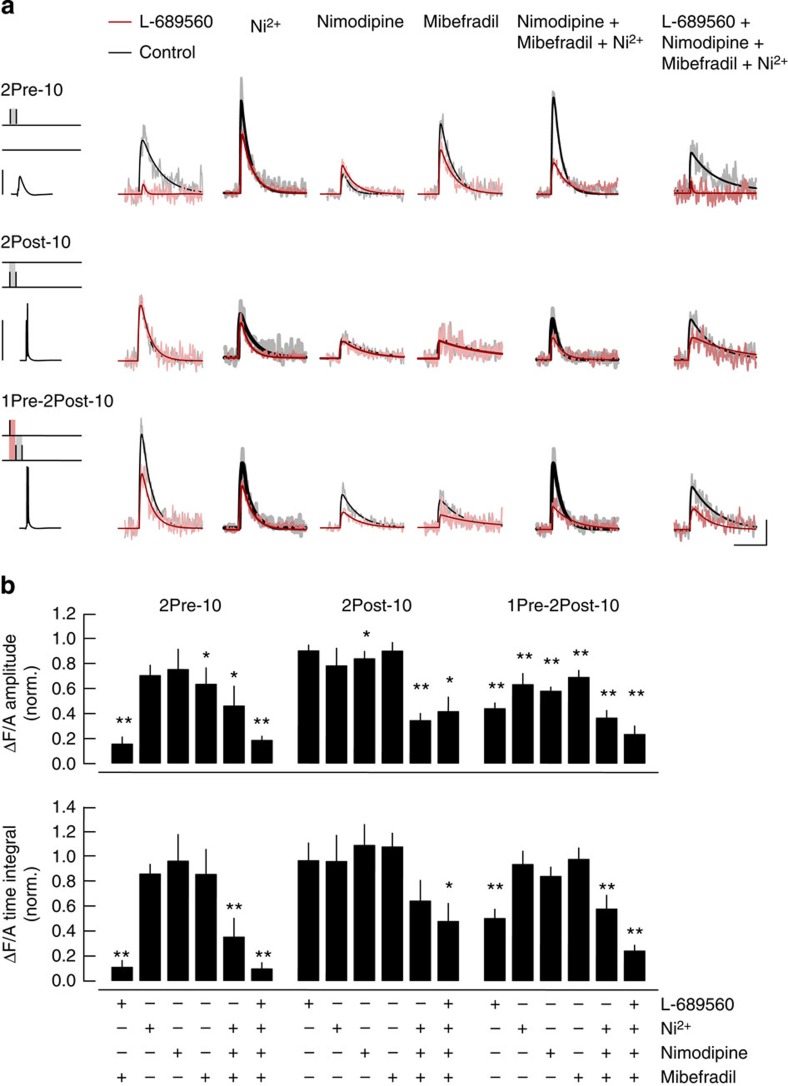
EPSCaTs evoked during paired stimulations are initiated through the activation of NMDARs and VSCCs. (**a**) Spine EPSCaTs evoked by 2Pre-10 (top), 2Post-10 (middle) or the LTP-inducing stimulation 1Pre-2Post-10 (bottom) before (control, black) and during bath application of antagonists (red): L-689560 (5 μM, *n*=10 spines, 5 cells), NiCl_2_ (50 μM, *n*=16 spines, 5 cells), Nimodipine (20 μM, *n*=12 spines, 6 cells), Mibefradil (5 μM, *n*=15 spines, 8 cells), VSCC antagonists cocktail alone (Mibefradil: 5 μM, Nimodipine 20 μM and NiCl_2_: 50 μM; *n*=9 spines, 4 cells) and with L-689560 (5 μM, *n*=7 spines, 3 cells). Left: schematics of the stimulus layout, and recorded somatic membrane potentials beneath. Right: EPSCaT waveforms (average of 6–7 trials) overlaid with fitted exponential rise and decay curves. Scale bars, horizontal: 0.1 s; vertical: 0.05 ΔF/A and 50 mV (10 mV for 2Pre-10). (**b**) Summary of EPSCaT amplitude (top) and time integral (bottom) in the presence of the antagonists in **a**, normalized to the control values before drug application. *P<0.05; **P<0.01, Wilcoxon rank-sum test.

To assess the relative contribution of NMDARs and VSCCs to spine Ca^2+^ transients elicited by the paired stimulations, we recorded individual spine EPSCaTs elicited by three interleaved stimulation protocols (2Pre-10, 2Post-10 and 1Pre-2Post-10) before (control) and during bath application of antagonists (Fig. 5). The NMDAR antagonist L-689560 abolished the EPSCaTs elicited by presynaptic paired pulses, and strongly inhibited the spine Ca^2+^ transients triggered by the LTP-inducing stimulation, while having no effect on the Ca^2+^ signals triggered by the bAPs. Individual VSCC type-selective antagonists inhibited the Ca^2+^ transient triggered by the 2Pre-10, 2Post-10 and 1Pre-2Post-10 stimulations to various degrees without any one antagonist showing substantial reduction. However, the VSCC antagonist cocktail substantially reduced the Ca^2+^ transient amplitude for the 2Post-10 and 1Pre-2Post-10 stimulations (Fig. 5 and Supplementary Tables 1 and 2). Presynaptic R-type VSCCs have been shown to control neurotransmission at the hippocampal mossy fibre–CA3 synapse, but the data supporting a role for R-, T- or L-type VSCCs at SC synapses in CA1 is less clear^51^,^52^,^53^. We controlled for this possibility by measuring the summated EPSPs evoked by 2Pre-10 stimulations and found no effect of the VSCC antagonists. EPSP peak amplitudes (mV) during control and antagonist application, were, respectively, for L-689560: 10.98±1.18 and 9.34±1.13 (*P*=0.39); Mibefradil: 12.26±1.19 and 15.91±3.87 (*P*=0.86); Nimodipine: 12.63±1.12 and 13.25±1.53 (*P*=0.69), NiCl_2_: 13.70±0.90 and 13.53±1.08 (*P*=1); Nimodipine+Mibefradil+NiCl_2_: 10.88±1.68 and 10.78±1.46 (*P*=1); L-689560+Nimodipine+Mibefradil+NiCl_2_: 11.87±1.00 and 9.72±1.23 (*P*=0.2).

Collectively, these results indicate that the activation of synaptic NMDAR and of spine VSCCs have a significant role in the spine Ca^2+^ transients during STDP. Together with our observations from the plasticity experiments, these results confirm that NMDAR-mediated Ca^2+^ influx is required for LTP and further suggest that, although the Ca^2+^ influx through each individual VSCC type alone is not required; the combined influx through two or more types of VSCC at dendritic spines is a critical component of LTP induction.

### Role of mGluRs in STDP

Postsynaptic membrane depolarization by bAPs coincident with synaptic release at the excitatory synapses on CA1 pyramidal cells can increase the lifetime of glutamate in the synaptic cleft^54^. At cerebellar granule cell synapses this phenomenon boosts the activation of perisynaptic group I metabotropic glutamate receptors (mGluRs) to facilitate NMDAR currents^55^. Group I mGluRs can also increase dendritic Ca^2+^ by stimulating release from Ca^2+^ stores via IP3 receptors^56^. Functional evidence for the role of mGluRs in synaptic plasticity at CA1 pyramidal cells^57^,^58^,^59^ suggests that the pre–post spike coincidence may also engage this mechanism at Schaffer collateral-CA1 synapses.

**Figure 6:**
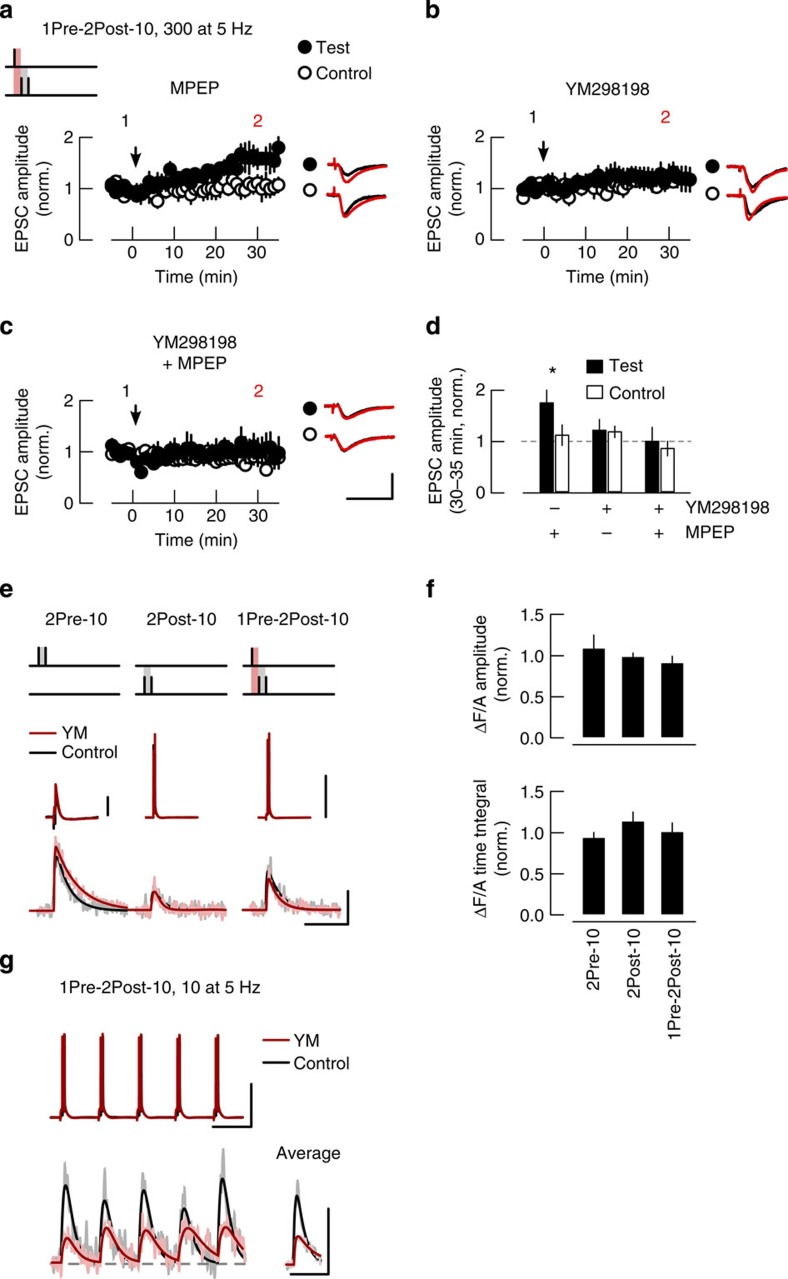
Induction of LTP by STDP requires the activation of mGlu1 receptors during the theta train. (**a**–**c**) 1Pre-2Post-10 stimulations induce test pathway-specific LTP in the presence of MPEP (10 μM, *n*=6), but not in the presence of YM298198 (YM 100 nM, *n*=7) or MPEP and YM298198 (*n*=5). Drugs were bath-applied throughout the experiment. Panels depict the time course of EPSC amplitude in test and control pathways, normalized to 5 min average before plasticity induction (arrows). Insets: average EPSC waveforms over 5 min before (1) and 30–35 min after (2) plasticity induction. Scale bars in **a**–**c**, 50 pA and 50 ms. (**d**) Summary of the changes in normalized EPSC amplitude at 30–35 min after plasticity induction. (**e**) EPSCaTs evoked by 2Pre-10, 2Post-10, or 1Pre-2Post-10 stimulations before drug application (control, black) and in the presence of YM298198 (100 nM, *n*=15 spines, 7 cells, red) overlaid with double exponential fits. (**f**) Summary of EPSCaT amplitude and time integral in the presence of YM298198, normalized to control before drug. (**g**) YM298198 attenuates the EPSCaTs evoked during a 2-s train of 1Pre-2Post-10 stimulations at 5 Hz. Top: somatic membrane potential; bottom: EPSCaTs recorded during the last second of the stimulus train (left) and their average waveforms (right). Traces are averages of 6–8 trials before (black) and during YM298198 application (red). Scale bars in **e** and **g**, horizontal: 0.2 s; vertical: 0.05 ΔF/A and 50 mV (10 mV for 2Pre-10).

Therefore we next tested whether group I mGluRs are required for LTP induction. The mGluR5 selective antagonist 2-methyl-6-(phenylethynyl)pyridine hydrochloride (MPEP; 10 μM) had no effect on LTP induction by a train of 1Pre-2Post-10 stimulations (Fig. 6a,d). In contrast, block of mGluR1 by the selective antagonist YM298198 (100 nM) or combined block of both mGluR1 and mGluR5 abolished the induction of LTP (Fig. 6b–d). These results show that the induction of LTP by 1Pre-2Post-10 stimulation train is dependent on the activation of mGluR1 subtype.

To investigate the mechanism by which mGluRs facilitate LTP induction we tested whether mGluRs directly facilitate Ca^2+^ transients by recording EPSCaTs evoked by individual stimulations as described above for the VSCC antagonists. YM298198 (100 nM) had no effect on the EPSCaTs triggered by either 2Pre-10, 2Post-10, or 1Pre-2Post-10 (Fig. 6e,f). mGluR1s are coupled to a Gq G-protein/phospholipase C (PLC) signalling pathway that is at least one order of magnitude slower than the fast ion channel-initiated EPSCaTs^56^. Thus the effects of mGluR1 activation might be expected to develop slowly during the course of an LTP-inducing stimulus train. We tested this hypothesis by imaging spine EPSCaTs during a short theta frequency train of 1Pre-2Post-10 stimulations (Methods section and Supplementary Fig. 3a). Individual EPSCaTs decayed within 200 ms and therefore did not summate during a 5-Hz LTP-inducing stimulus train (Supplementary Fig. 3a and Fig. 6g). YM298198 (100 nM) reduced the amplitude of the EPSCaTs evoked during the stimulus train (compared with control before antagonist : 0.6±0.11, *P*<0.05; *n*=9 spines, 4 cells, Fig. 6g), with no effect on the time integrals (0.83±0.17, *P*=0.35, compared with control). This attenuation was comparable to that of Ca^2+^ transients elicited by 1Pre-2Post-10 in the presence of VSCC antagonist cocktail (*P*=0.16, two-sided unpaired Wilcoxon rank-sum test), that also inhibited LTP. Furthermore, during the stimulus train we did not observe a sustained rise in spine Ca^2+^ that was sensitive to YM298198. Together, these results indicate that LTP induction requires mGluR1 activation during coincident pre- and postsynaptic activity that increases the Ca^2+^ influx during repeated presentations of coincident activity.

**Figure 7:**
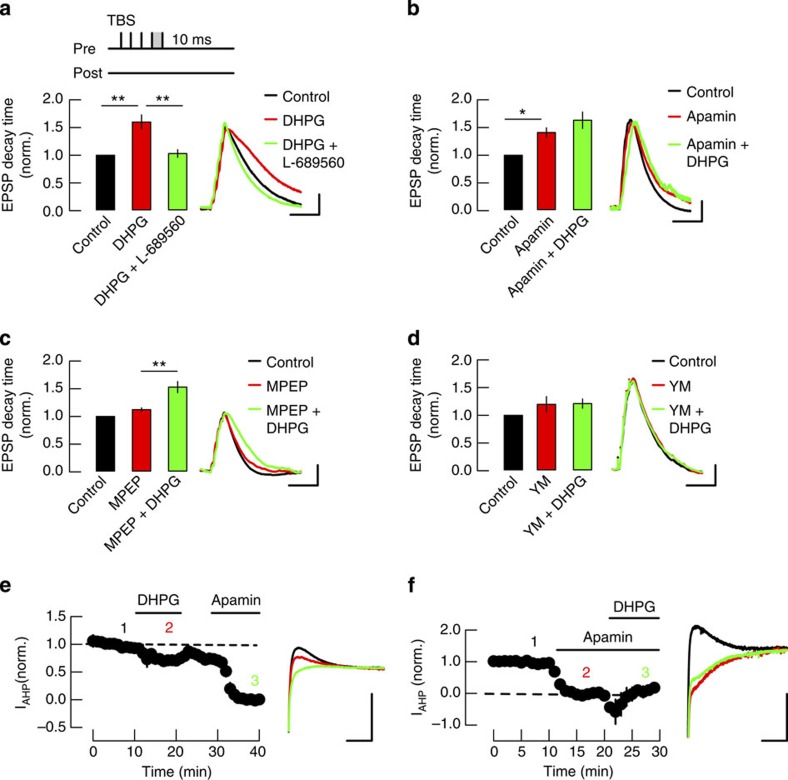
mGluR1 receptors prolong the NMDAR-mediated component of EPSPs through the inhibition of SK channels. (**a**) DHPG (50 μM) prolongs the duration of summated EPSPs evoked by five presynaptic stimulations at 100 Hz (TBS) and the effect is reversed by 5 μM L-689560 (5 μM, *n*=7). (**b**) Apamin (100 nM) prolongs the EPSPs and occludes the effect of DHPG (*n*=7). (**c**) MPEP (30 μM) does not affect EPSP prolongation induced by DHPG (*n*=8). (**d**) YM298198 (1 μM) blocks the prolongation of EPSPs induced by DHPG (*n*=8). Bar graphs in **a**–**d** show the average EPSP decay time constant normalized to the values recorded before drug application (control). Insets: example of peak-normalized EPSP traces. Scale bars in **a**–**d**, 2 mV and 0.1 s. (**e**) DHPG (50 μM) or apamin (100 nM) depress the I_AHP_ currents recorded from CA1 pyramidal cells in perforated patch clamp (*n*=6). Time course of I_AHP_ amplitude after subtraction of apamin-insensitive component and normalization to control before drug wash in. Insets: example I_AHP_ traces in control condition (1, black), during DHPG (2, red) and apamin (3, green) application. Scale bars, 50 pA and 0.1 s. (**f**) Apamin (100 nM) occludes the effect of DHPG (50 μM) on I_AHP_ (*n*=5). Time course of apamin-sensitive I_AHP_ amplitude normalized to control. Insets: example I_AHP_ traces in control condition (1, black), during apamin (2, red) and apamin plus DHPG (3, green). Scale bars in **e** and **f**, 50 pA and 0.1 s. *P<0.05; **P<0.01, Wilcoxon rank-sum test.

Our next goal was to determine the mechanism by which mGluR1 facilitates Ca^2+^ influx. Group I mGluRs are coupled to the Gq G-protein that activates phospholipase C signalling pathway causing release of Ca^2+^ from internal stores. However, this mechanism would be expected to induce a slowly developing and persistent release of Ca^2+^ (ref. 56), which we did not observe. Interestingly, group I mGluRs activate a signalling pathway in common with the M1 muscarinic receptors (mAChR) that have been shown to facilitate NMDAR function via the inhibition of a Ca^2+^-dependent SK channel-mediated negative feedback loop^60^,^61^. We therefore investigated whether this mechanism is also engaged by mGluR1. NMDAR modulation by SK channels is most physiologically relevant and clearly demonstrated at resting membrane potentials under normal extracellular Mg^2+^ (ref. 60) so similar to previous studies we used the decay time constant of summated EPSPs evoked by a burst of presynaptic stimuli in slices from juvenile rats as a measure of the NMDAR component of the synaptic response^60^,^62^ (Methods section). Bath application of the group I mGluR selective agonist (*S*)-3,5-dihydroxyphenylglycine (DHPG; 50 μM) depolarized the membrane potential by 3.4±2.1 mV and prolonged the summated EPSPs without affecting EPSP amplitude (Fig. 7a). This effect was reversed by addition of the NMDAR antagonist 5 μM L-689560 (Fig. 7a). In separate experiments, L-689560 (5 μM) alone did not affect the EPSP decay time (normalized to control: 0.9±0.04, *P*=0.06, *n*=5) confirming previous observations^60^,^62^. When the membrane potential was repolarized to the membrane potential before DHPG, L-689560 still reduced the EPSP decay time constant, indicating the enhancement of the NMDAR-mediated component of the EPSP was not due to membrane depolarization (decay time constant normalized to control 1.35±0.15 in DHPG and 0.78±0.11 with addition of L-689560, *n*=7, *P*<0.01). The selective SK channel blocker apamin (100 nM) also increased the EPSP decay time constant and occluded the effect of 50 μM DHPG (Fig. 7b). The effects of DHPG were not affected by 30 μM MPEP (Fig. 7c) but were blocked by 1 μM YM298198 (Fig. 7d). The effects of DHPG and apamin on the decay time constant of summated EPSPs were replicated in slices from mature rats demonstrating the regulation of NMDAR activity by mGluRs was consistent regardless of developmental stage (Supplementary Fig. 6). These results show that activation of mGluR1 facilitates NMDAR activation and indicates that the mechanism is via inhibition of SK channels.

**Figure 8:**
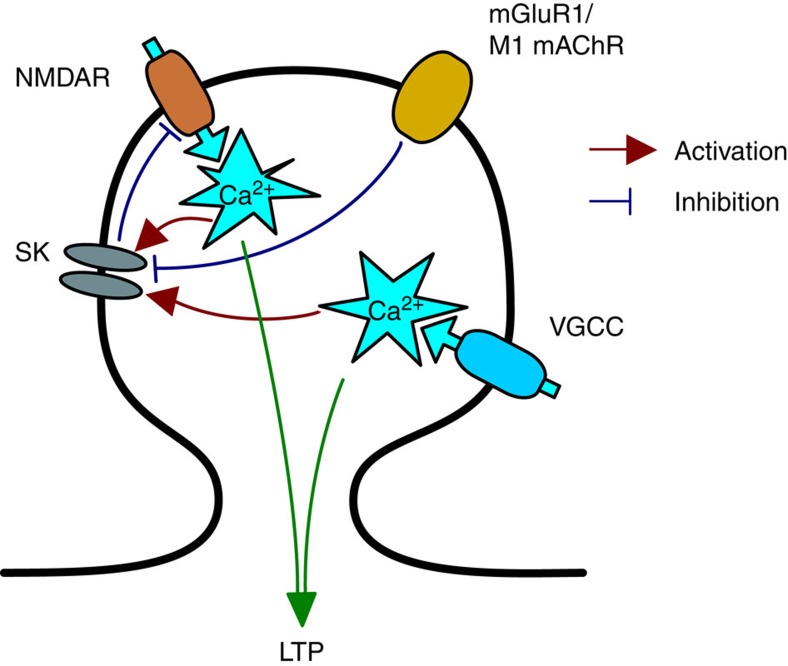
Spine Ca^2+^ transients are controlled by mGluR1 or M_1_ muscarinic receptor inhibition of SK channels. mGluR1 or M_1_ muscarinic receptors inhibit SK channels, which removes a negative feedback regulation of NMDARs within dendritic spines thereby enhancing Ca^2+^ influx. Facilitated Ca^2+^ influx through NMDARs and Ca^2+^ influx through VSCCs are both required for the induction of LTP by STDP.

To test this directly we measured SK channel activity by recording afterhyperpolarization currents (I_AHP_) in voltage clamp using the perforated patch technique (Methods section). Membrane depolarization from −50 to +10 mV for 100 ms and back to −50 mV in the presence of the KCNQ channel blocker XE-991 (10 μM) revealed an I_AHP_ that was inhibited by apamin (100 nM; Fig. 7e,f) allowing subsequent pharmacological subtraction of the SK-mediated component of the I_AHP_. DHPG (50 μM) depressed the SK-mediated I_AHP_ (Fig. 8e; normalized to control before drug: 0.8±0.08, *P*<0.05; *n*=6) that partially recovered on washout. DHPG (50 μM) had no effect on I_AHP_ after application of 100 nM apamin confirming that DHPG inhibited SK-mediated currents (Fig. 7f; *n*=7). These results show that mGluR1 activation facilitates NMDAR function by inhibiting SK channels, likely by acting via the G-protein/PLC signalling in common with M_1_ mAChRs.

## Discussion

In this study we have identified distinct Ca^2+^ sources that are required for the induction of LTP by precisely timed pre- and postsynaptic spiking at mature hippocampal synapses. We also show that the combined Ca^2+^ signal from these sources does not predict plasticity outcome and that mGluR1 activation is required for LTP. From these observations we propose that postsynaptic spiking has a central role in the induction of STDP by providing activation of VSCCs, depolarization for NMDARs and indirect enhancement of NMDAR function by facilitating mGluR1 activation. Thus, postsynaptic spiking is a better predictor of LTP induction than Ca^2+^ influx through NMDARs.

Our test of the Ca^2+^ hypothesis predictions showed that EPSCaTs measured across the entire spine head did not correlate with the induction of STDP. In particular, EPSCaTs in response to paired presynaptic stimulation (2Pre-10) were consistently larger than EPSCaTs produced by any other STDP protocol including those that induced LTP. This suggests that the membrane potential within spines is substantially depolarized by EPSPs and that high levels of NMDAR activation can be produced by EPSPs in the absence of bAPs. Large amplitude spine EPSPs can only be generated if the spine neck resistance is sufficiently large to provide a high degree of electrical isolation of spine heads from the parent dendrites. Indeed this has been demonstrated by voltage dye, EPSCaT and super-resolution imaging studies coupled with electrophysiological recording and modelling^21^,^22^,^63^. Together, these observations argue that bursts of presynaptic stimulation produce substantial Ca^2+^ influx through NMDARs but since presynaptic bursts alone do not induce synaptic plasticity, Ca^2+^ influx through NMDARs cannot be the only trigger for synaptic plasticity. In this study we show that another critical trigger for the induction of synaptic plasticity is the activation of VSCCs.

L-, R- and T-type VSCCs have all been shown to contribute to Ca^2+^ transients in dendritic spines^23^,^47^. Our data broadly support these conclusions and show that combined blockade of these three VSCC types reduces EPSCaTs evoked by paired pre- and postsynaptic stimulation, whereas blockade of each subtype alone only contributes a small component. As expected, EPSCaTs evoked by presynaptic stimulation were mediated primarily by NMDARs, whereas EPSCaTs evoked by postsynaptic bAPs were mediated principally by VSCCs. A significant fraction of the presynaptically evoked EPSCaTs when NMDARs were active was mediated by VSCCs (Fig. 5). These EPSCaTs were almost entirely blocked by the NMDAR antagonist, suggesting that NMDAR activation during presynaptic bursts may provide enough spine membrane depolarization to activate VSCCs. Interestingly, a component of EPSCaTs evoked by bAPs was not mediated by L-, R- or T-type VSCCs that could potentially result from N- or P/Q-type channels. EPSCaTs evoked by paired pre- and postsynaptic stimulation were mediated by both NMDARs and VSCCs supporting the requirement for both Ca^2+^ sources for LTP induction. Furthermore, LTP was only prevented by blockade of L-, R- or T-type VSCCs together but not each subtype separately indicating that individual VSCC subtypes may each contribute to a common Ca^2+^ influx required for LTP. This may explain why previous studies have found no or only partial inhibition of STD–LTP after blockade of individual VSCC subtypes^3^,^28^,^49^.

An additional role for postsynaptic spikes in the induction of synaptic plasticity comes from the observation that postsynaptic depolarization prolongs the dwell time of glutamate in the synaptic cleft at central synapses including hippocampal Schaffer collateral-CA1 (ref. 54). In the cerebellum, this promotes the recruitment of perisynaptic group I mGluRs to facilitate the NMDAR-mediated component of the synaptic response via a fast, Homer-mediated direct action on NMDARs^55^. We also find that recruitment of mGluR1 facilitates NMDAR activity but show that this is a slow indirect mechanism since blockade of mGluR1 only reduces EPSCaTs evoked by multiple rather than isolated paired stimulations. Furthermore, our data do not show a sustained increase in basal Ca^2+^ during repetitive pre- and postsynaptic paired stimulation arguing against a role for IP3 receptor-mediated release of Ca^2+^ from internal Ca^2+^ stores. We propose that mGluR1 enhances NMDAR activation by a similar mechanism to that engaged by another Gq coupled receptor M_1_ muscarinic receptors^60^,^61^; namely that mGluR1 inhibits Ca^2+^-sensitive SK channels which normally function to restrict NMDAR activation as part of a negative regulatory feedback mechanism within dendritic spines^47^,^64^ (Fig. 8). Our data support a model where the requirement for mGluRs in synaptic plasticity is to facilitate NMDAR activity which may not be necessary under strong induction protocols^57^,^58^,^59^,^65^.

Contrary to the canonical rules for STDP at immature synapses there is a notable absence of LTD induced by anti-causal pairings of pre- and postsynaptic spikes in our data and other studies of STDP at mature synapses^32^,^33^,^66^. STD–LTD at immature synapses is not dependent on postsynaptic NMDARs and instead requires postsynaptic mGluR and VSCC activation leading to endocannabinoid production, which retrogradely reduces presynaptic release probability^28^,^29^,^30^,^31^. Mature synapses apparently lack this form of LTD induction. We show LTD occurs at mature hippocampal synapses but it requires prolonged low-frequency presynaptic stimulation and the amplitude of EPSCaTs produced by this stimulation does not map onto the Ca^2+^ hypothesis for STDP (Fig. 3). These results argue for a reassessment of the rules defining STDP at mature hippocampal synapses. Spike timing is important and controls the induction of LTP but anti-causal spike timings do not induce LTD.

Here we show that Ca^2+^ influx to the spine through both NMDARs and VSCCs is required for the induction of STD–LTP. Coupled with the lack of correlation between EPSCaT magnitude and LTP induction, this suggests there are distinct Ca^2+^ sensors located in nanodomains surrounding the Ca^2+^ sources separated by the presence of Ca^2+^ buffers^25^,^26^,^27^. In addition, evidence indicates that Ca^2+^ signals from these sources can be augmented by Ca^2+^-induced Ca^2+^ release from internal stores that are present in a subset of spines^41^,^67^,^68^. Moreover, Ca^2+^ elevations in the spine heads are short-lived owing to locally controlled plasma membrane extrusion, sequestration in the internal stores, and diffusion through the spine neck^19^,^20^,^23^,^69^. An important, yet still unanswered question is how these mechanisms converge onto distinct Ca^2+^ sensors to shape the spine response to pre-and postsynaptic activity patterns and determine the direction and magnitude of Hebbian synaptic plasticity.

## Methods

### Slice preparation

Acute hippocampal slices were prepared from adult (P50-55, 200–250 g) male Wistar rats following a lethal dose of isoflurane inhalation, in accordance with Home Office guidelines as directed by the Home Office Licensing Team at the University of Bristol. For experiments on summated EPSPs and I_AHPs_ (Fig. 7) juvenile (p13–15) male Wistar rats were used similar to previous studies^60^. Hippocampi were dissected in ice-cold slicing solution containing (in mM): 119 NaCl, 10 glucose, 26.2 NaHCO_3_, 2.5 KCl, 1 NaH_2_PO_4_, 0.5 CaCl_2_ and 5 MgCl_2_, 300 mOsm, equilibrated with 95% CO_2_ and 5% O_2_ then mounted on agar. Transverse slices were cut (400-μm thick) using a VT1200 vibratome (Leica). Slices were incubated in artificial cerebrospinal fluid (aCSF) containing (in mM): 119 NaCl, 10 glucose, 26.2 NaHCO_3_, 2.5 KCl, 1 NaH_2_PO_4_, 2.5 CaCl_2_ and 1.3 MgCl_2_, 300 mOsm, equilibrated with 95% CO_2_ and 5% O_2_ at 36 °C for 30 min, then kept at room temperature until use. For synaptic plasticity experiments, the slices were cut between CA3 and CA1 just before being transferred to the recording chamber.

### Electrophysiology

Whole-cell patch-clamp recordings were made from CA1 pyramidal neurons visualized under infrared differential interference contrast on a SliceScope Pro 6000/Multiphoton Imaging System (Scientifica) in a recording chamber superfused (∼1.5–2 ml min^−1^) with aCSF at 35 °C containing 50 μM picrotoxin to block GABA_A_ receptor-mediated transmission. Patch electrodes (3–5 MΩ) were pulled from borosilicate filamented glass capillaries (Harvard Apparatus) on a PC-87 Micropipette Puller (Sutter Instrument). Electrodes were filled with intracellular solution containing (in mM): 117 KMeSO_3_, 8 NaCl, 1 MgCl_2_, 10 HEPES, 0.2 EGTA, 4 MgATP and 0.3 Na_2_GTP, buffered to pH 7.2, 280 mOsm.

Recordings were made with a Multiclamp 700A amplifier (Molecular Devices), filtered at 4 kHz and digitized at 10 kHz using a CED micro 1401 MKII board and Signal 5 acquisition software (Cambridge Electronic Design). Synaptic responses were evoked using 0.1–1 ms square pulses (Digitimer). When used, back-propagated postsynaptic action potentials (bAPs) were elicited via somatic current injection (1–2 nA, 2 ms). Membrane voltage was not corrected for liquid junction potential, which was calculated at −9 mV.

For STDP experiments, synaptic responses were recorded in voltage clamp (−70 mV). EPSCs were evoked alternatively at 0.1 Hz in test and control pathways using tungsten bipolar stimulating electrodes (100 kΩ, 119 μm tip spacing, MicroProbes) placed in the stratum radiatum, on opposite sides of the patched cell and at different distances from the stratum pyramidale. The pathways were tested regularly for independence using paired-pulse protocols. Consecutive EPSCs were averaged online every minute, and their amplitudes were normalized offline to the average of 5 min before the plasticity induction protocol (baseline). The relative change in synaptic strength (Fig. 3b) was calculated as the difference between the average change in EPSC amplitudes in the test and control pathway over the last 5 min of recording, relative to baseline. Series resistance was monitored throughout the experiments and cells with series resistance above 30 MΩ or showing >20% change were discarded from subsequent analysis. Spike timing and theta burst pairing protocols were applied in current-clamp mode within 10 min of establishing whole-cell configuration to avoid washout of LTP. The average resting membrane potential of recorded CA1 pyramidal cells was −73.3±2.2 mV (*n*=52 cells without drug treatment). For spike timing protocols, the test pathway received a theta frequency train (300 stimulations at 5 Hz for 1 min) unless specified otherwise. EPSPs and summated EPSPs were set to be subthreshold for action potential generation. The pre–post timing intervals were measured between the onsets of the presynaptic stimulus and that of the first postsynaptic spike (Fig. 1). The theta burst pairing protocol consisted of three trains of 10 bursts. Each burst consisted of five coincident pre- and postsynaptic spikes at 100 Hz. The frequency of the bursts was 5 Hz. The trains were separated by 10 s intervals. When used, drugs were bath-applied throughout the experiment.

Summated EPSPs recorded in the presence of GABA_A_ and GABA_B_ receptor antagonists picrotoxin (50 μM) and CGP55849 (5 μM) were evoked by a burst of stimuli consisting of five subthreshold stimuli at 100 Hz every 30 s and three consecutive sweeps were averaged every minute. EPSP decay times were determined by fitting average EPSP waveforms with a single exponential decay function between peak and return to resting membrane potential. Drugs were washed in after 10 min recording (control) and applied for 10 min. Decay times in the presence of drugs were normalized to the mean control values and averaged over the last 5 min of drug application.

I_AHP_ currents were recorded in voltage clamp using the perforated patch technique. Perforated patch recording were performed using patch pipettes (5–6 MΩ tip resistance) tip filled with intracellular solution containing (in mM): 120 KMeSO_3_, 8 NaCl, 10 KCl, 10 HEPES, 0.2 EGTA, 4 MgATP and 0.3 Na_2_GTP, buffered to pH 7.4, 280 mOsm and then back filled with the same solution supplemented freshly with 80 μg ml^−1^ gramicidin from dimethylsulphoxide stock solution (20 mg ml^−1^). After forming a gigaohm seal, the series resistance was monitored and recordings commenced once it stabilized usually 30–40 min after seal formation. Average series resistance for perforated patch recordings was ∼40 MΩ. Spontaneous rupture of the perforated patch was checked by continuous monitoring of the series resistance. I_AHPs_ were elicited by a 100 ms depolarizing step from −50 to +10 mV then back to −50 mV, every 30 s. Consecutive traces were averaged every minute and the amplitude was calculated as the difference between the I_AHP_ peak and the current before the step depolarization. Drugs were applied for 10 min after the control period and the I_AHP_ amplitude values over the last 5 min were averaged.

### Two-photon Ca^2+^ imaging

For spine Ca^2+^ imaging the intracellular solution was supplemented on the day of the experiment with a Ca^2+^ fluorescent indicator (Fluo-5F, 200 μM) and a reference fluorescent dye (Alexa Fluor 594, 30 μM) from stock solutions. The medium affinity Ca^2+^ dye Fluo-5F was chosen for its better dynamic range and reduced buffer capacity compared with commonly used dyes OGB-1 and Fluo 4 but has low fluorescence at rest necessitating the use of a reference fluorescent dye^70^. To avoid additional Ca^2+^ buffer capacity EGTA was omitted from the intracellular solution, which contained (in mM): 117 KMeSO_3_, 8 NaCl, 1 MgCl_2_, 10 HEPES, 4 MgATP, and 0.3 Na_2_GTP, buffered to pH 7.2, 280 mOsm. Spine Ca^2+^ transients (EPSCaTs) were imaged on secondary apical dendrites in dual-channel fluorescence^40^ via a × 60 water immersion objective (Olympus). Fluorescence excitation source was provided by a Ti:sapphire laser (Newport Spectra-Physics) tuned to 810 nm. Whole-cell configuration was initially established in voltage clamp (−70 mV). Cells were switched to current clamp and dye-loaded by injecting 100–150 pA inward current for 10–15 min. Subthreshold EPSPs were evoked at 0.1 Hz via a monopolar extracellular patch electrode filled with 5 μM Alexa-containing aCSF for visualization (Fig. 2a). The electrode was placed in the stratum radiatum in the proximity of the patched cell. Spines were initially visualized in raster scanning mode to allow for the placement of the stimulating electrode tip at ∼20–30 μm distance from an apical dendritic branch. Optically responsive spines were located as described previously^40^. EPSCaTs were recorded in line scanning mode. Line-scan series (1,000 lines per second for 1 s) were acquired with a single spike timing stimulation (see above) delivered 0.25 s after the start of each series. To minimize photodamage EPSCaTs were recorded in batches of up to nine line-scan series, every 15–20 s. Different spike timing stimulations were interleaved within a batch. EPSCaT traces were then grouped by stimulation type and averaged offline. Where drugs were applied, data from batches acquired 5 min after drug wash in were normalized to the data collected before drug application (control). Imaged spines were monitored for drift in the Alexa channel and drifts <1 μm were compensated. Cells were discarded from subsequent analysis whenever the imaged spines or parent dendrites had localized swelling, sustained increase in the resting Ca^2+^ fluorescence, or when the stimulation resulted in a tonic increase in Ca^2+^ fluorescence without return to pre-stimulus level. Resting membrane potential was continuously monitored and cells with resting membrane potentials above −60 mV or changes >10 mV were discarded. Images (12 bit quantization) were acquired with a data acquisition board (National Instruments Corporation) using ScanImage r3.8 software.

Fluorescence data were denoised using the PURE-LET algorithm then EPSCaT traces were calculated as the relative change in Fluo-5F fluorescence channel versus Alexa Fluor channel (ΔF/A) and fitted with a double exponential function (Fig. 2a)^40^. EPSCaTs elicited by paired stimulations with ISI of 50 ms (2Pre-50, 2Post-1Pre-50, and 1Pre-2Post-50, Fig. 2b) could be resolved in a doublet of Ca^2+^ transients 50 ms apart and were fitted with a sum of two double exponential functions (blue and green traces in Fig. 2b). We used the peak amplitude of the fitted curve (ΔF/A amplitude) and the time-integrated change in Ca^2+^ fluorescence (ΔF/A time integral) as EPSCaT measures throughout. ΔF/A time integral was calculated by numerical integration of the fitted function over 0.5 s interval from the onset of the transient. Fluorescence data were not calibrated for Ca^2+^ concentration.

For theta train EPSCaT recordings, short trains of EPSP–2bAP pairs (1Pre-2Post-10 stimulations, at 5 Hz for 2 s) were delivered and spines were imaged during the second half of the stimulus train, to avoid photodamage. Ca^2+^ transients were time-locked with the stimulus, and had variable intensity consistent with the occurrence of EPSCaTs evoked by stochastic neurotransmitter release summated with bAP-elicited Ca^2+^ transients (Supplementary Fig. 3). The acquisition was repeated 6–8 times (20 s interval) in two batches: before (control) and 5 min after drug wash in. Data within a batch were averaged then EPSCaT traces were fitted with the sum of five exponential rise and decay curves separated by 0.2 s, and the waveform average of the five EPSCaTs was calculated.

### Analysis of optical response potencies

Successes and failures in optical response were discriminated by comparing the signal energy in the Fluo-5F signal (the square root of the sum of squared signal values) during the putative EPSCaT peak (50 ms after the stimulus) to that of the baseline (50 ms before stimulus), termed the *l*_2_ norm ratio^40^. Responses were deemed successful if the peak to baseline *l*_2_ norm ratio was at least 1.3, as detailed previously^40^. For 50 ms ISI stimulations, two putative EPSCaT peak windows were used to test for each Ca^2+^ transient in the doublet. The average EPSCaT response to a given stimulation type was determined as the average of all trials, including failures. The EPSCaT potency was defined as the size (peak amplitude, time integral) of the average successful response elicited by a single stimulus or jointly by the components of a paired stimulation. For single pre- or postsynaptic stimuli, EPSCaT potency was determined as the average of successful trials. For paired stimulation with 50 ms ISI, the EPSCaT potency was determined as the average of the trials successful in both components of the Ca^2+^ transient doublet. The potency of EPSCaTs evoked by paired stimulations with 10 ms ISI was inferred using the approach described below.

We considered four possible outcomes when two independent stimuli *S*_1_ and *S*_2_ are delivered as a pair (*S*_1_*S*_2_): (1) no response, with probability *P*_00_; (2) response only to *S*_1_, with probability *P*_10_; (3) response only to *S*_2_, with probability *P*_01_; (4) joint response (evoked by both *S*_1_ and *S*_2_), with probability *P*_11_. Furthermore, *P*_p_ is the probability of a successful optical response (any of the outcomes 2–4). These probabilities are related by equations:


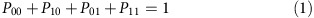



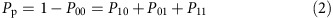


When *S*_1_ or *S*_2_ are delivered individually, EPSCaTs occur as Bernoulli trial processes with probability *P*_1_ or *P*_2_, respectively. In particular, single EPSPs and single bAPs elicit spine Ca^2+^ transients stochastically^41^,^42^. Assuming that the responses to *S*_1_ and *S*_2_ are independent, *P*_01_ and *P*_10_ can be expressed as the success probability of one stimulus conditioned on a failure of the other:


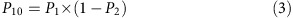



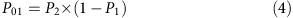


We also denote by *A*_1_ and *A*_2_ the response potencies of *S*_1_ and *S*_2_ when tested separately, and by *A*_p_ the potency of the response to *S*_1_*S*_2_ with any of the outcomes 2–4. Assuming linear summation of the responses, the joint potency *A*_11_ can be calculated as:


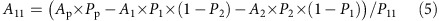


The unknown probabilities *P*_1_, *P*_2_ and *P*_p_ were estimated by their corresponding sample frequencies of success 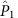, 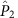 and 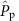 determined experimentally. The potencies *A*_1_, *A*_2_ and *A*_p_ were determined as the average of optically successful responses to *S*_1_, *S*_2_ and *S*_1_*S*_2_, respectively. When used, the postsynaptic spike pairs at 100 Hz were considered as a unique stimulus. For the 2Pre-10 stimulations, the probability 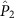 of success for the second EPSP was further adjusted by a paired-pulse facilitation previously described at the Schaffer collateral-CA1 synapses^36^:


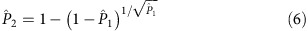


Linearity factors for the summation of EPSCaTs elicited by paired pre- and postsynaptic stimulations were calculated as the ratio of the EPSCaT potency normalized to the sum of the EPSCaT potencies of individual stimuli. The analysis was performed offline with software written in Matlab (The MathWorks). Distances from the imaged spines to the soma were measured using the Simple Neurite Tracer plugin for Fiji/ImageJ on image stacks acquired in the Alexa channel at the end of the experiment. For illustration purposes, raster scan (XY) images were noise filtered with a three-dimensonal median filter. The cell overview in Fig. 2 was obtained from three partially overlapping image Z stacks that were collated using Pairwise stitching plugin for Fiji/ImageJ.

### Statistical analysis

Statistical analysis was performed in R. Data distributions were tested for normality using Lilliefors (Kolmogorov-Smirnov) test. Significant differences between the Ca^2+^ responses to different stimulations were tested at the family level using one-way ANOVA, followed by multiple pairwise comparisons with Tukey HSD test. The EPSCaTs were also compared directly to the 2Pre-10 responses using Dunnett *post hoc* adjustment for many-to-one comparisons. Statistical comparisons for normalized data were performed using two-sided Wilcoxon rank-sum tests. Sample sizes were determined by power calculations based on typical effect sizes. For synaptic plasticity experiments comparisons were made between the test and control pathways on EPSC amplitudes averaged over the last 5 min of recording against the null hypothesis of no difference between sample means. Linear correlations were tested against the hypothesis of no correlation between data sets. Other statistical tests were performed as described in Results section. The level of significance was set to 0.05. The calculated probabilities are symbolized by asterisks as follows: *P<0.05, **P<0.01, ***P<0.001. Pooled data are presented as mean±s.e.m.

### Reagents

Picrotoxin, ethylene glycol-bis(2-aminoethylether)-*N*,*N*,*N*′,*N*′-tetraacetic acid (EGTA), trans-2-Carboxy-5,7-dichloro-4-phenylaminocarbonylamino-1,2,3,4-tetrahydro-quinoline (L-689560), Nimodipine, Mibefradil, apamin, TTX, DHPG, MPEP and 6-amino-*N*-cyclohexyl-*N*,3-dimethylthiazolo[3,2-a]benzimidazole-2-carboxamide (YM298198) hydrochloride were purchased from Tocris. Fluo-5F and Alexa Fluor 594 were purchased from Invitrogen. All other chemicals were purchased from Fisher Scientific.

## Additional information

**How to cite this article:** Tigaret, C. M. e*t al.* Coordinated activation of distinct Ca^2+^ sources and metabotropic glutamate receptors encodes Hebbian synaptic plasticity. *Nat. Commun.* 7:10289 doi: 10.1038/ncomms10289 (2016).

## Supplementary Material

Supplementary Information Supplementary Figures 1-6 and Supplementary Tables 1-2
