# Supplementary material for: Coordinated activation of distinct Ca2+ sources and metabotropic glutamate receptors encodes Hebbian synaptic plasticity
Source: Nat Commun. 2016 Jan 13;7:10289. doi: 10.1038/ncomms10289 (PMC4735496; doi:10.1038/ncomms10289)
Supplement: Supplementary Information — Supplementary Figures 1-6 and Supplementary Tables 1-2 [file ncomms10289-s1.pdf]

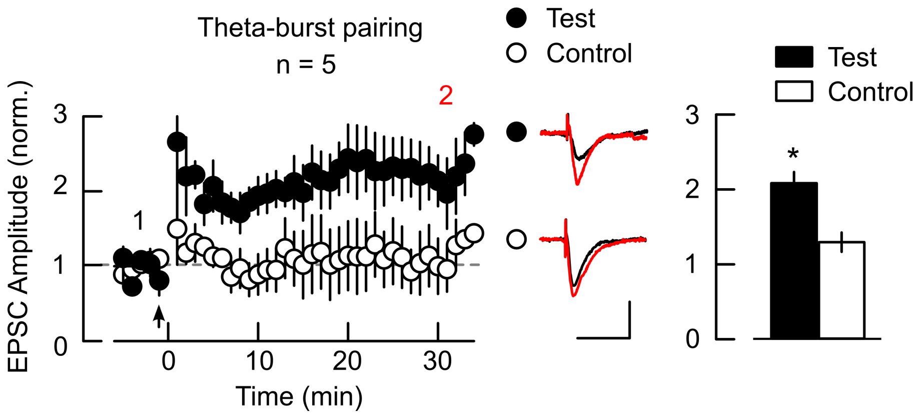

Supplementary Figure 1: LTP induction in adult slices at CA3 – CA1 synapses by theta-burst pairing protocol. *Left*: time course of EPSC amplitude in Test and Control pathways; values are normalized to last 5 min of baseline before induction protocol as applied to the Test pathway (arrow). *Middle*: Average EPSC waveforms taken over 5 min during the baseline (1) and 30 – 35 min after TBS (2). Scale bars: 50 pA and 50 ms. *Right*: Summary of the values at 30 – 35 min.

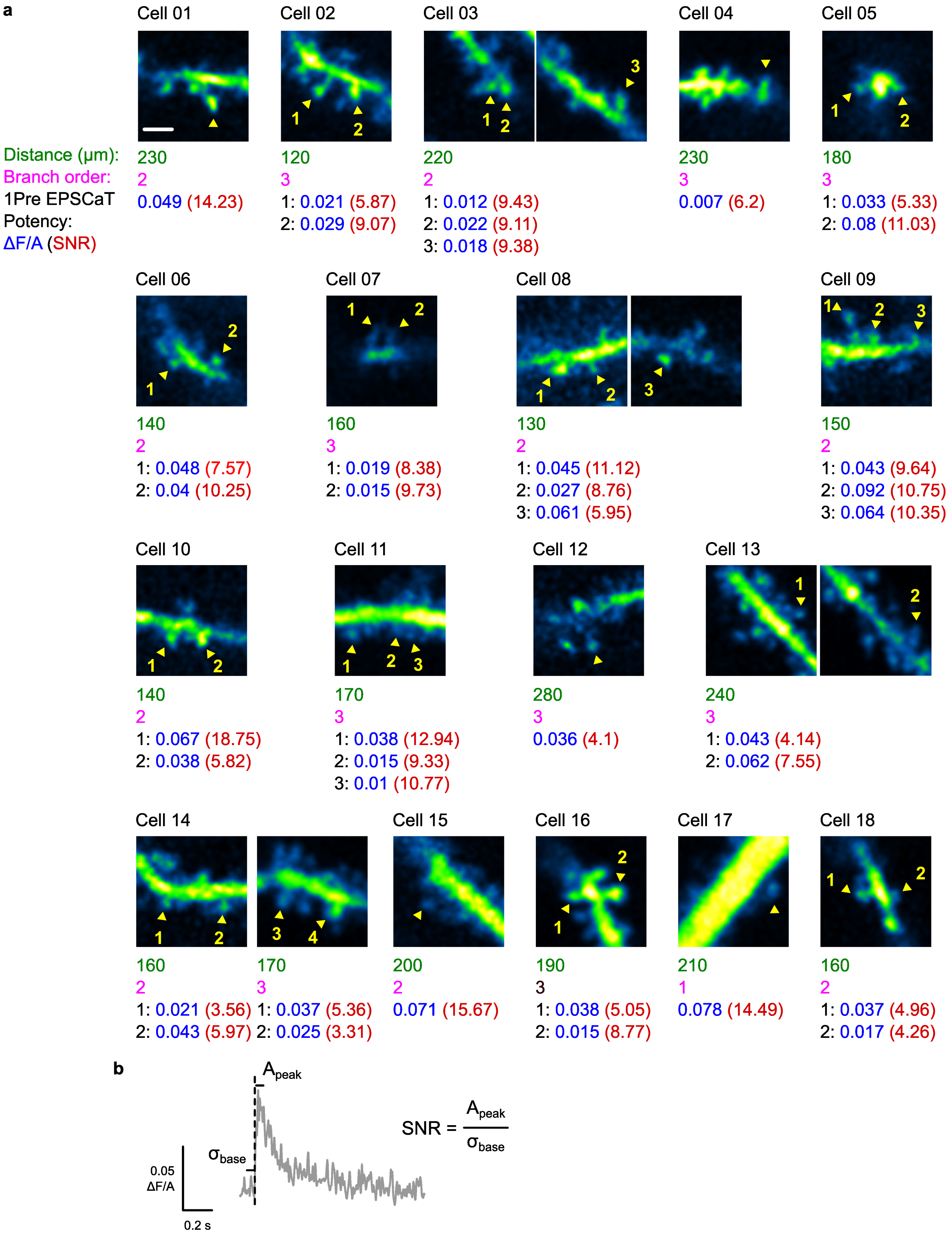

Supplementary Figure 2: Distribution of Ca^{2+} responses across the spines sampled in Figure 2 and Supplementary Fig. 4. **a.** Distribution of spines used in analysis visualized in the Alexa fluorescence channel. Arrowheads and numbers indicate optically responsive spines to 1Pre stimulations. Scale bar: 2 μm. Distance from soma (green), dendrite branch order (pink) and EPSCaT amplitude for 1Pre stimulation are all indicated for each spine. EPSCaT amplitude is expressed in ΔF/A (blue) and signal-to-noise ratio (SNR, red) **b.** SNR values in a were calculated as the ratio between the average value of the Fluo-5F signal during the putative EPSCaT peak (50 ms after the stimulus indicated by the vertical dashed line) and the standard deviation of the baseline (50 ms before the stimulus).

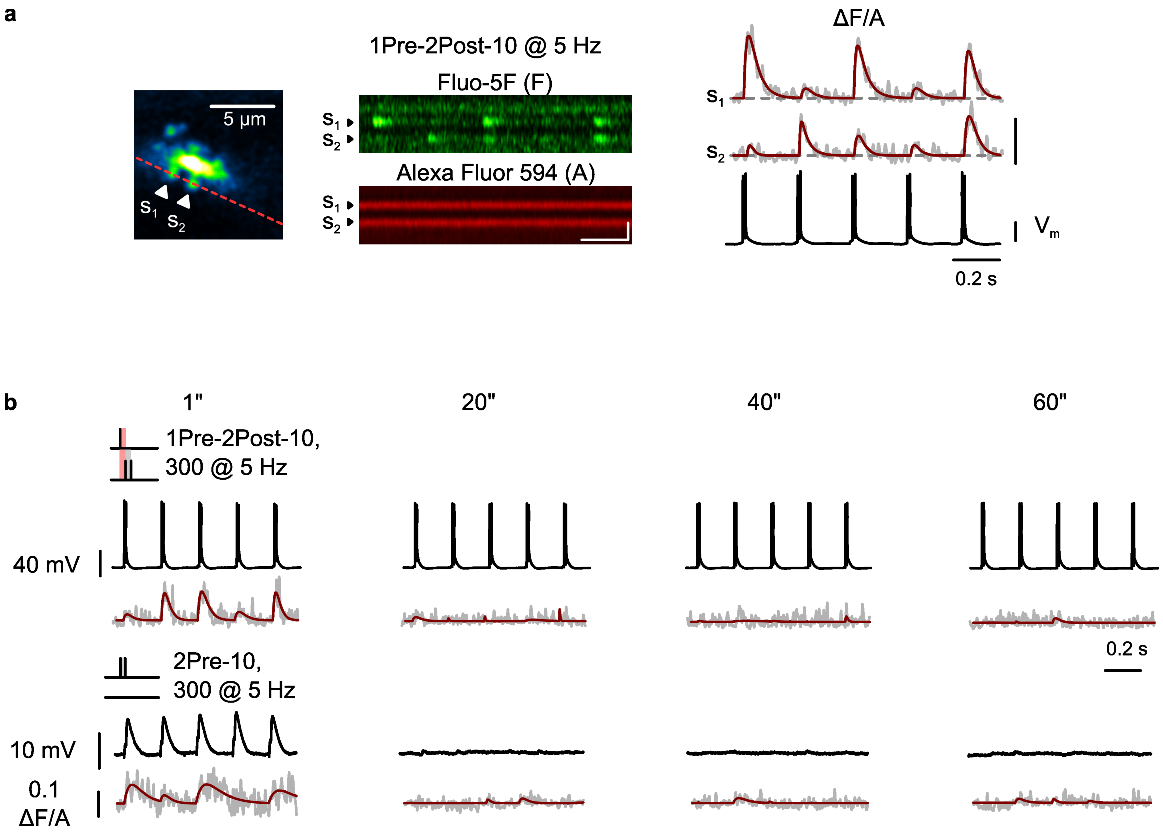

Supplementary Figure 3: EPSCaTs evoked by pre- and postsynaptic spike pairings delivered as theta trains. **a.** EPSCaTs do not summate during stimulus trains. EPSCaTs are asynchronously evoked in neighbouring spines by a theta (5 Hz) train of 1Pre-2Post-10 stimulations. *Left*: XY raster scanning image (Alexa Fluor channel) of two spines (r1, r2) imaged for EPSCaTs in line scanning mode (dashed red line). Scale bar: 5 μm). *Middle*: Dual fluorescence line-scan series (Fluo-5F and Alexa Fluor) acquired in the spines shown at *Left* during the theta train. Individual EPSCaTs are detected in the Fluo-5F channel (green) time-locked with the stimulus. *Right*: EPSCaT traces (gray) recorded in spines r1 and r2, fitted with a sum of delayed exponential rise and decay curves (red); somatic membrane potentials (Vm) are depicted below. Scale bars: 0.2 s, 2 μm for the linescan series, 0.1 ΔF/A for the EPSCaTs, and 50 mV for the membrane potential. **b.** EPSCaTs attenuate during the first 20 seconds of theta trains. Membrane voltage recorded at the soma (black) and spine EPSCaTs (gray) evoked during the first, 20th, 40th and 60th second (denoted “) of 1Pre-2Post-10 or 2Pre-10 theta trains (300 stimulations at 5 Hz for 1 min). EPSCaT traces were fitted with a sum of five exponential rise and decay curves separated by 200 ms (red).

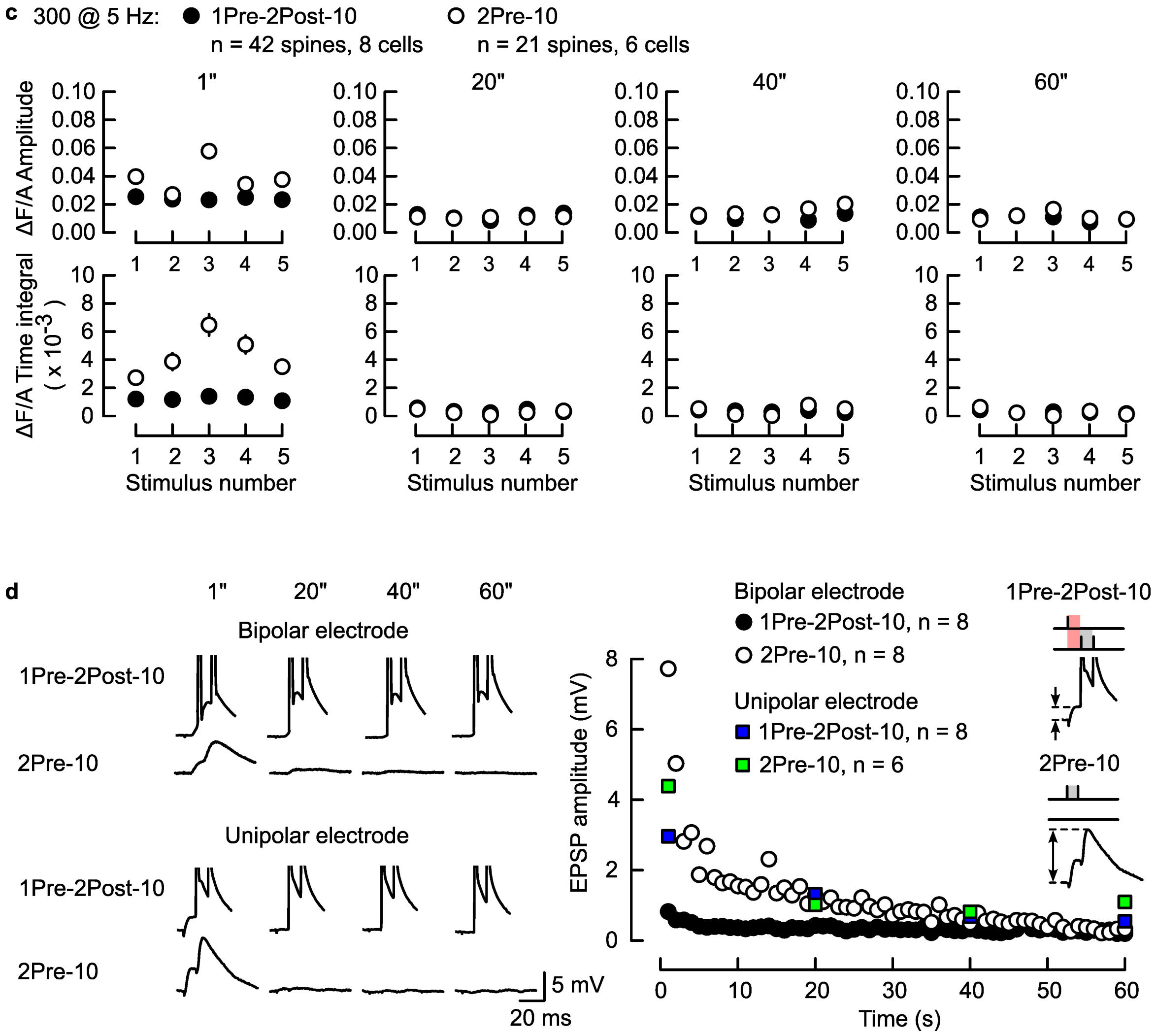

Supplementary Figure 3: (continued). **c.** Summary of amplitudes (top) and time integrals (bottom) of individual EPSCaTs during 1Pre-2Post-10 (n = 42 spines, 8 cells) and 2Pre-10 (n = 21 spines, 6 cells) theta trains, recorded at the time indicated above the plots. Data shown as mean ± SEM. **d.** EPSPs attenuate during trains evoked by either a tungsten bipolar electrode or a local unipolar glass electrode. *Left*: Representative examples (average of 5 successive responses) of somatic membrane voltage recorded at 1, 20, 40 and 60 seconds during a theta train. Responses to 1Pre-2Post-10 stimulations are scaled up and action potential waveforms clipped to reveal the synaptically-evoked component. *Right*: Time course of the EPSP amplitude (1 s average) measured as illustrated in the insets (bipolar 1Pre-2Post-10: n = 8 cells and 2Pre-10: n = 8 cells; unipolar 1Pre-2Post-10: n = 8 cells and 2Pre-10, n = 6 cells, mean ± SEM). Bipolar electrode data was recorded during plasticity induction for the experiments in Figure 1 c,e, main text.

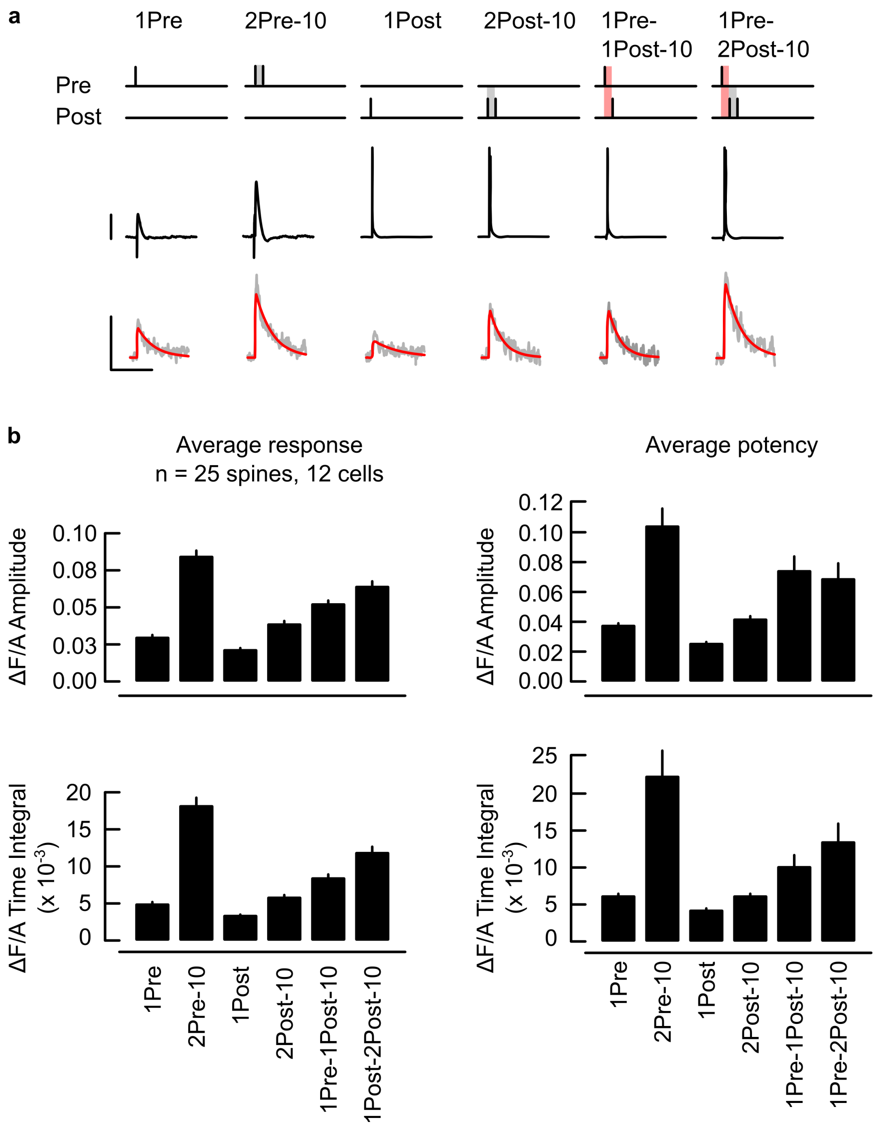

Supplementary Figure 4: Magnitude of spine EPSCaTs elicited by pre- and postsynaptic spike pairing.
a. EPSCaTs evoked by pairing pre- and postsynaptic spikes. *Top*: schematics of presynaptic input and postsynaptic spikes within individual stimulations (time intervals as in Figure 1). *Middle*: Somatic membrane potential waveforms. *Bottom*: EPSCaT waveforms averaged over 7 – 9 trials evoked by the stimulations depicted at the *top* (black) overlaid with fitted exponential rise and decay curves (red). Scale bars, horizontal: 0.1 s; vertical: 50 mV (10 mV for 1Pre and 2Pre-10) and 0.05 ΔF/A. **b.** Summary of EPSCaT amplitudes and time integrals (left: average responses; right: average potencies, see Methods) for the stimulations in **a**.

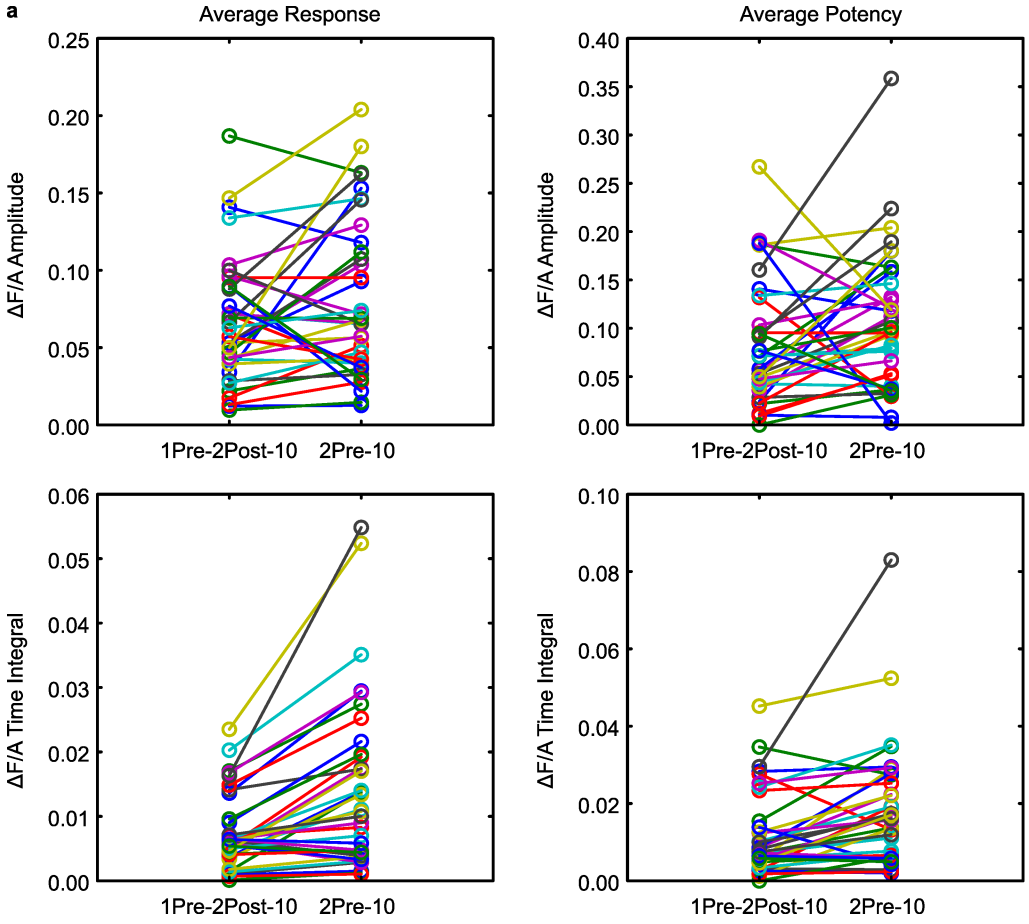

Supplementary Figure 5: Lack of correlation between LTP and EPSCaT magnitude does not depend on spine size or potency.
a. EPSCaTs elicited by 1Pre-2Post-10 (LTP-inducing) are smaller than those triggered by 2Pre-10 (non-LTP-inducing) in nearly all spines analysed (one spine corresponds to a pair of data points) regardless of EPSCaT magnitude. Values are average of all trials including failures (*left*) or average potencies (*right*).

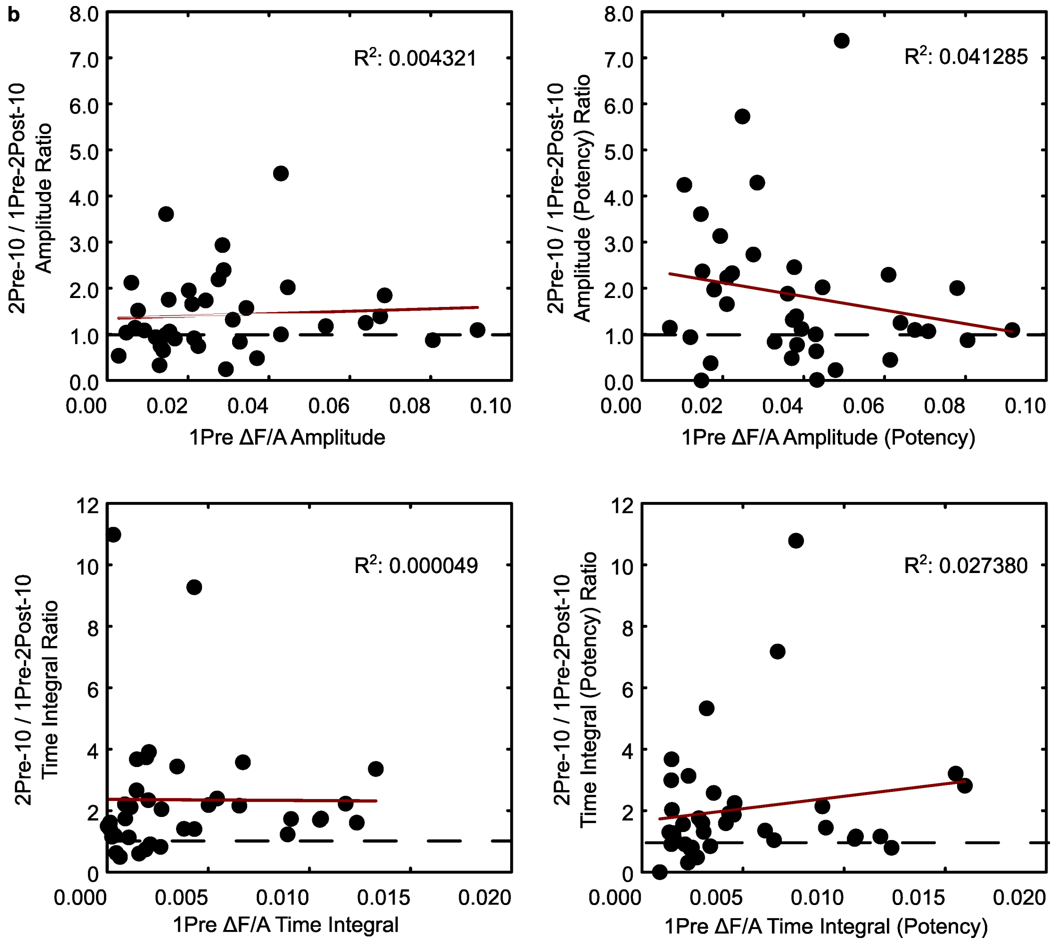

Supplementary Figure 5: (continued). **b.** There is no correlation between EPSCaT magnitude and the ratio of EPSCaTs elicited by 2Pre-10 compared to 1Pre-2Post-10 across a full range of EPSCaT magnitudes in individual spines (amplitude and time integral ratios, linear regression shown in dark red). Horizontal dashed lines indicate a ratio of 1. *Left*: Average values across all trials including failures. *Right*: Average potencies (see Methods). Spines in **a** and **b** belong to the data sets in Figure 2 and Supplementary Fig. 4.

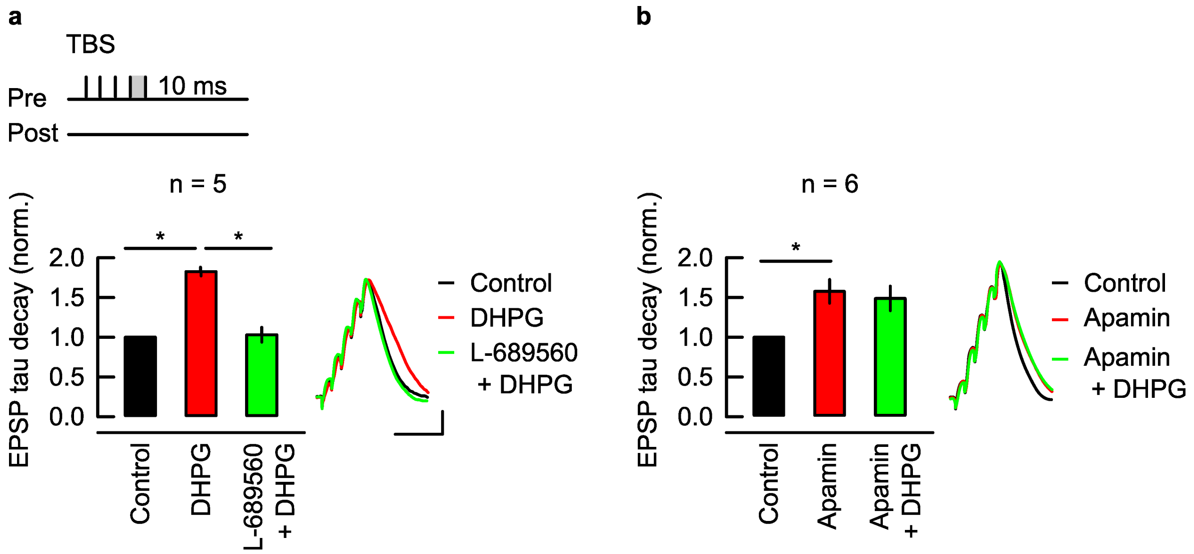

Supplementary Figure 6: mGluR1 receptors prolong NMDAR-dependent EPSPs via inhibition of SK channels in adult hippocampal CA1 pyramidal neurons.

a. DHPG (50 μM) increases the decay time constant of summated EPSPs evoked by five presynaptic stimuli at 100 Hz (TBS). This effect is reversed by 5 μM L-689560 (n = 5). **b.** Apamin (100 nM) prolongs the TBS-evoked EPSPs and occludes the effect of DHPG (n = 6). Bar graphs in **a** and **b** show the average EPSP decay time constant normalized to values recorded before drug wash-in (Control). *Insets*: example of peak-normalized EPSP traces. Scale bars: 2 mV and 0.1 s.

Supplementary Table 1: Effects of NMDAR and VSCC antagonists on EPSCaT amplitude.
EPSCaT amplitude under NMDAR and VSCC antagonists, normalized to the values before drug application (Control). Data given as mean ± SEM and p-values for two-tailed Wilcoxon rank sum tests against the null hypothesis that the true mean equals 1.

**Stimulation Protocol**	**L-689560 5 μM**	**$NiCl_2$ 50 μM**	**Nimodpine 20 μM**	**Mibefradil 5 μM**	**Nimodipine +Mibefradil +$NiCl_2$**	**L-689560 +Nimodipine +Mibefradil +$NiCl_2$**
	**10 spines 5 cells**	**16 spines 5 cells**	**12 spines 6 cells**	**15 spines 8 cell**	**9 spines 4 cells**	**7 spines 3 cells**
**2Pre-10**	0.15 ± 0.05<br>$p < 0.01$	0.78 ± 0.13<br>$p = 0.09$	0.75 ± 0.16<br>$p = 0.12$	0.63 ± 0.13<br>$p < 0.05$	0.45 ± 0.15<br>$p < 0.05$	0.18 ± 0.03<br>$p < 0.01$
**2Post**	0.89 ± 0.04<br>$p = 0.06$	0.83 ± 0.11<br>$p = 0.06$	0.83 ± 0.05<br>$p < 0.05$	0.89 ± 0.06<br>$p = 0.13$	0.34 ± 0.05<br>$p < 0.01$	0.41 ± 0.11<br>$p < 0.05$
**1Pre-2Post-10**	0.43 ± 0.04<br>$p < 0.01$	0.70 ± 0.08<br>$p < 0.01$	0.57 ± 0.03<br>$p < 0.001$	0.68 ± 0.05<br>$p < 0.001$	0.36 ± 0.06<br>$p < 0.01$	0.23 ± 0.06<br>$p < 0.01$

Supplementary Table 2: Effects of NMDAR and VSCC antagonists on EPSCaT time integrals.
Table shows the time integrals for EPSCaTs recorded during antagonist application normalized to the values before drug application (mean ± SEM and p-values for two-tailed Wilcoxon rank sum tests against the null hypothesis that the true mean equals 1). Drug concentrations and sample sizes as in Supplementary Table 1.

**Stimulation Protocol**	**L-689560**	**$NiCl_2$**	**Nimodpine**	**Mibefradil**	**Nimodipine +Mibefradil +$NiCl_2$**	**L-689560 +Nimodipine +Mibefradil +$NiCl_2$**
**2Pre-10**	0.11 ± 0.05<br>$p < 0.01$	0.95 ± 0.20<br>$p = 0.21$	0.96 ± 0.21<br>$p = 0.79$	0.85 ± 0.2<br>$p = 0.26$	0.35 ± 0.15<br>$p < 0.01$	0.10 ± 0.05<br>$p < 0.01$
**2Post**	0.96 ± 0.14<br>$p = 0.76$	0.93 ± 0.10<br>$p = 0.25$	1.08 ± 0.17<br>$p = 1$	1.07 ± 0.11<br>$p = 0.64$	0.64 ± 0.17<br>$p = 0.07$	0.48 ± 0.14<br>$p < 0.05$
**1Pre-2Post-10**	0.50 ± 0.08<br>$p < 0.01$	0.85 ± 0.07<br>$p = 0.07$	0.84 ± 0.07<br>$p = 0.06$	0.97 ± 0.09<br>$p = 0.71$	0.58 ± 0.11<br>$p < 0.01$	0.24 ± 0.05<br>$p < 0.01$
